# Computational Architectures for Precision Dairy Nutrition Digital Twins: A Technical Review and Implementation Framework

**DOI:** 10.3390/s25164899

**Published:** 2025-08-08

**Authors:** Shreya Rao, Suresh Neethirajan

**Affiliations:** 1 Faculty of Computer Science, Dalhousie University, 6050 University Avenue, Halifax, NS B3H 4R2, Canada; sh316686@dal.ca; 2 Faculty of Agriculture, Dalhousie University, Truro, NS B3H 4R2, Canada

**Keywords:** digital twin, precision dairy nutrition, livestock monitoring, edge computing in agriculture, hybrid modeling, sensor fusion, smart farming, sustainable livestock systems, real-time data integration

## Abstract

Sensor-enabled digital twins (DTs) are reshaping precision dairy nutrition by seamlessly integrating real-time barn telemetry with advanced biophysical simulations in the cloud. Drawing insights from 122 peer-reviewed studies spanning 2010–2025, this systematic review reveals how DT architectures for dairy cattle are conceptualized, validated, and deployed. We introduce a novel five-dimensional classification framework—spanning application domain, modeling paradigms, computational topology, validation protocols, and implementation maturity—to provide a coherent comparative lens across diverse DT implementations. Hybrid edge–cloud architectures emerge as optimal solutions, with lightweight CNN-LSTM models embedded in collar or rumen-bolus microcontrollers achieving over 90% accuracy in recognizing feeding and rumination behaviors. Simultaneously, remote cloud systems harness mechanistic fermentation simulations and multi-objective genetic algorithms to optimize feed composition, minimize greenhouse gas emissions, and balance amino acid nutrition. Field-tested prototypes indicate significant agronomic benefits, including 15–20% enhancements in feed conversion efficiency and water use reductions of up to 40%. Nevertheless, critical challenges remain: effectively fusing heterogeneous sensor data amid high barn noise, ensuring millisecond-level synchronization across unreliable rural networks, and rigorously verifying AI-generated nutritional recommendations across varying genotypes, lactation phases, and climates. Overcoming these gaps necessitates integrating explainable AI with biologically grounded digestion models, federated learning protocols for data privacy, and standardized PRISMA-based validation approaches. The distilled implementation roadmap offers actionable guidelines for sensor selection, middleware integration, and model lifecycle management, enabling proactive rather than reactive dairy management—an essential leap toward climate-smart, welfare-oriented, and economically resilient dairy farming.

## 1. Introduction

Digital transformation has emerged as a prominent global trend, spurring innovations across numerous technological domains. Among these, digital twin (DT) technology is particularly notable, having garnered significant interest due to its transformative capabilities [1,2,3,4,5]. A digital twin represents a dynamic, virtual counterpart of a physical entity or system, sustained by continuous, real-time data integration and exchange between its digital and physical dimensions.

Originally pioneered within manufacturing and aerospace industries, the digital twin concept has rapidly expanded into diverse sectors, including agriculture, healthcare, urban planning, and smart infrastructure. More than a conventional simulation, a digital twin serves as an adaptive, data-driven model, dynamically mirroring real-world conditions to facilitate real-time monitoring, predictive analytics, and informed decision-making. This paper presents a PRISMA-based systematic literature review, aiming to identify and critically compare computational architectures used in digital twins for dairy cows. While commercial development is still merging, this study synthesizes academic, industrial, and conceptual developments with a special focus on architectures applicable to commercial dairy farms.

**Figure 1:**
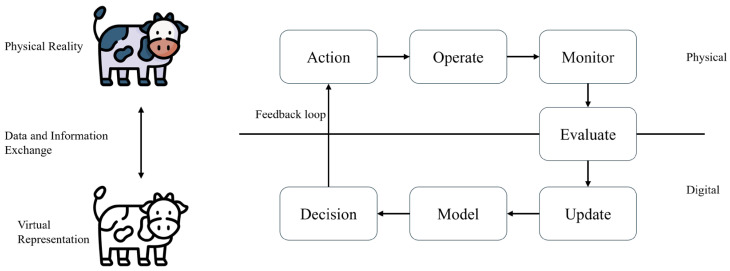
A high-level schematic illustrating the fundamental components and processes of a digital twin (DT) system in dairy nutrition applications. The diagram depicts the continuous data exchange between real-world sensors capturing animal and environmental data and the virtual modeling environment that facilitates predictive simulations, real-time analytics, and informed decision-making.

Grieves and Vickers [6] initially defined DTs within the framework of product lifecycle management, highlighting their significance in virtual representation and closed-loop optimization. Extending this perspective, Kritzinger et al. [7] characterized digital twins as actively connected virtual replicas, distinct from traditional, static simulations due to their continuous bidirectional data exchange and concurrent evolution. Negri et al. [8] further clarified distinctions between conceptual models, digital shadows, and true digital twins, asserting that authentic twins necessitate synchronized, real-time data streams coupled with feedback mechanisms. Figure 1 depicts a general digital twin architecture adapted specifically to dairy farming applications, illustrating sequential data flow from sensor-based data acquisition through preprocessing, computational modeling, and simulation, culminating in actionable decision-making and iterative system refinement.

In agriculture, particularly dairy farming, digital twins are increasingly investigated for real-time modeling of animal behavior, metabolic dynamics, and precision feeding. However, few existing deployments satisfy the rigorous technical and practical criteria necessary for robust commercial implementation.

### 1.1. Evolution of Digital Twin Technology Across Domains

The digital twin paradigm has significantly evolved over the last two decades, shifting from offline simulation tools toward intelligent, autonomous systems. Originally conceptualized at NASA to manage spacecraft systems, DTs have since become foundational to Industry 4.0, underpinned by the integration of IoT sensors, big data analytics, AI, and edge computing.

**Figure 2:**
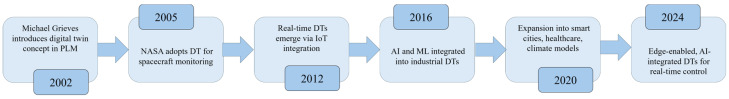
Historical evolution of digital twin technology from initial conceptualizations in 2002 through progressive advancements to 2024. Key milestones indicate the transition from early offline simulations to contemporary edge-AI integrated, real-time predictive models, highlighting significant developments relevant to precision livestock systems.

In the early 2000s, DTs were primarily used for design-time and post-failure analysis. With the rise of virtual–physical systems, DTs transformed into real-time systems capable of predictive diagnostics, anomaly detection, and operational optimization. Jeong et al. [9] outlined this trajectory as the shift from “descriptive” to “prescriptive” digital twins. Wu et al. [10] described a layered framework, describing the evolution of technology ranging from data acquisition and connectivity to autonomy and self-optimization. The technological evolution [11] of digital twins from NASA’s early applications to AI-integrated edge systems is depicted in Figure 2, highlighting key milestones that underpin the paradigm’s transition from simulation to autonomy.

As DTs mature into intelligent systems, their adaptation to livestock applications requires rethinking their computational architecture to account for real-time biological variability, heterogeneous sources, and on-farm edge constraints. These challenges remain unexplored in the literature.

### 1.2. Importance of Digital Twins in Various Sectors

Digital twin systems are central to digital transformation strategies across many domains including agriculture, manufacturing, healthcare, and urban planning. Their ability to combine real-time sensing, simulation, and integration of artificial intelligence enables precise control and prediction in complex environments. While digital twin technology has gained popularity in healthcare, planning, and manufacturing, its application in agriculture remains underexplored and fragmented. This review concentrates on this emerging domain. DTs, especially in dairy systems, enable real-time monitoring of individual cows, personalized nutrition, and early disease prediction.

**Figure 3:**
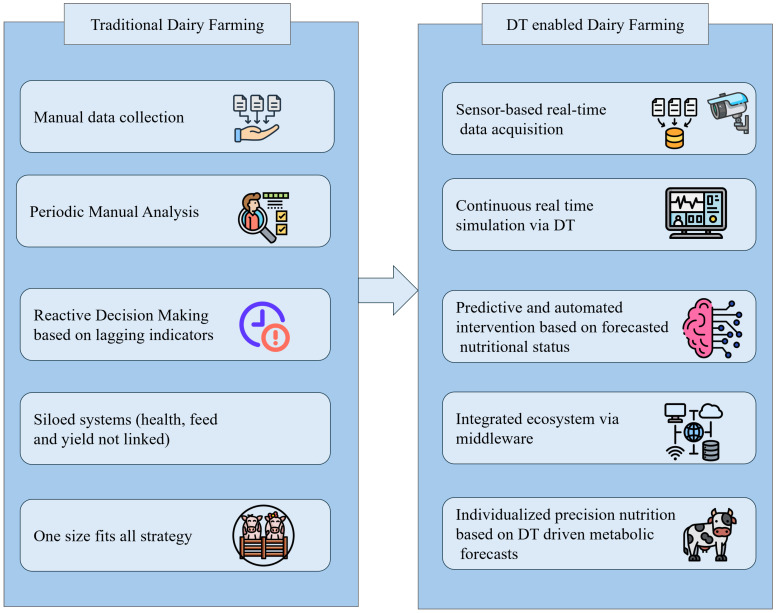
A comparative representation of traditional versus digital twin-enabled dairy farming systems. The diagram underscores how digital twins facilitate the shift from reactive, fragmented approaches towards integrated, proactive management strategies, enabling real-time monitoring, personalized nutrition interventions, and predictive decision-making.

In agriculture, DTs are used to model livestock behavior, optimize precision nutrition, and reduce greenhouse gas emissions. In dairy farming, DTs simulate individual cow physiology and behavior to personalize feeding and predict health issues [12]. Figure 3 contrasts traditional dairy farming with DT-enabled dairy farming, emphasizing how digital twins transform the system from reactive and siloed to proactive, integrated, and personalized. In manufacturing, DTs support predictive maintenance, fault detection, and real-time production optimization [13], while in healthcare, emerging applications range from personalized medicine to mental healthcare [14] such as PsyDT [15], which builds a digital twin of a psychological counselor using LLMs for adaptive counseling.

Jones et al. [16] categorized the DT application domains across sectors such a urban planning, manufacturing, and energy systems, and provided a structured framework to evaluate DT maturity in each based on real-time responsiveness, integration level, and decision automation. Similarly, Qi and Tao’s [17] research underscores the role of networking infrastructure, cybersecurity, and data interoperability in the effectiveness of a digital twin.

While these insights offer valuable architectural blueprints, their direct application to livestock and dairy systems requires careful adaptation. Precision dairy farming introduces unique challenges ranging from biological variability and physiological dynamics to fragmented on-farm connectivity and stringent ethical considerations related to animal welfare that demand a context-aware restructuring of existing DT frameworks.

### 1.3. Objectives of the Review

This review seeks to critically explore the current landscape of digital twin technology, with a particular focus on computational architectures, modeling techniques, and deployment frameworks for precision dairy nutrition and sustainable agriculture. It integrates technological foundations and sector-specific case studies from over 100 journal articles.

Research Questions:

1. What conceptual and architectural models define a digital twin across domains and how can they be tailored to livestock systems?
2. How are digital twins implemented in agricultural and livestock contexts, particularly for nutrition and health prediction?
3. What are the current technical limitations in dairy nutrition modeling?
4. What are the key technical, infrastructural, and ethical challenges in deploying digital twins on commercial dairy farms?
5. What future opportunities exist for integrating AI, edge computing, and simulation technologies into next-generation dairy digital twins?

The review aims to bring together the key technologies and frameworks that make these DT systems possible, compare how they are applied across different sectors, and highlight the major gaps, challenges, and future research priorities in implementing them. It provides a comprehensive synthesis of digital twin technologies, with an emphasis on computational design, modeling approaches, and deployment strategies tailored to precision nutrition in dairy cows.

## 2. Methodology: PRISMA Based Systematic Review Approach

This literature review paper follows a systematic methodology based on best practices. The goal was to identify, evaluate, and synthesize scholarly work relevant to the application of DTs in precision dairy nutrition.

### 2.1. Literature Classification Approach

The review adopts a structured methodology to critically evaluate the current state of digital twin technologies as applied to precision dairy nutrition. In contrast to the lifecycle-based classifications often used in industrial digital twin reviews, this work organizes the literature around computational and functional dimensions that directly impact the modeling, implementation, and real-time utility of dairy-focused digital twins. The classification framework was iteratively developed after reviewing the foundational literature across domains and then refined to align with the unique demands of dairy systems, particularly the need for modeling biological variability, feed behavior, and metabolic processes in livestock.

Five interrelated classification dimensions guided the analysis. First, studies were categorized by application domain, distinguishing between general agriculture, livestock-focused work, and specifically dairy-oriented applications. This ensured that conclusions drawn from industrial or crop-based DTs were not over-generalized to biologically dynamic systems like dairy cows. The second dimension of classification was the modeling approach, which grouped works based on whether they relied on physics-based models (like mechanistic digestion simulators), machine learning techniques (LSTMs for rumination detection), or hybrid approaches that integrate empirical and theoretical logic. The third dimension was computational architecture. This involved classifying systems according to their data infrastructure, including centralized cloud-based designs, modular layered systems, or edge-enabled frameworks designed for intermittent connectivity typical in rural farm environments. This classification was crucial to understand how real-time decision support scales across different dairy farm sizes. The fourth axis concerned validation methodology, differentiating between conceptual studies, simulation-only validations, and field-tested deployments with real-time farm data. Finally, studies were classified by implementation maturity: whether the proposed digital twin remained at the theoretical stage, was validated through prototypes, or had been implemented in working farm environments with full feedback integration.

By mapping each article to these five dimensions, the review creates a comparative framework that highlights technical gaps, architectural bottlenecks, and domain-specific trade-offs in the deployment of digital twin systems for precision nutrition in dairy cattle.

### 2.2. Search Strategy for Identifying Relevant Papers

The review followed a multi-phase process designed to ensure both comprehensive coverage as well as relevance to the review’s specific computational focus. The first stage involved a systematic search of academic databases, including IEEE Xplore, Scopus, SpringerLink, Web of Science, arXiv, and PubMed, with a search window spanning from January 2010 to early February 2025. Search queries were structured around combinations of terms such as “digital twin”, “livestock monitoring”, “precision dairy nutrition”, “cow behaviour modeling”, “feed optimization algorithms”, “sensor fusion in agriculture”, and “edge computing in farm environments”. Boolean search strings were constructed to combine key thematic terms, for example, (“digital twin” OR “DT”) AND (“livestock” OR “dairy” OR “precision feeding” OR “cow behavior” OR “rumination monitoring” OR “feed optimization”) AND (“sensor fusion” OR “IoT” OR “edge computing” OR “real-time system”). These queries were tailored slightly across databases based on syntax requirements and were refined iteratively to reduce noise while ensuring inclusivity across domains.

**Figure 4:**
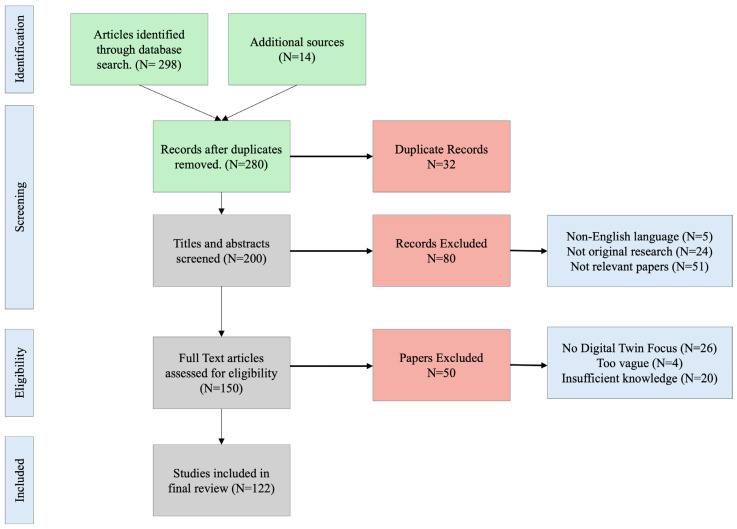
PRISMA (Preferred Reporting Items for Systematic Reviews and Meta-Analyses) 2020 flow diagram summarizing the methodological approach used in this systematic review. It outlines the literature identification, screening, eligibility assessment, and inclusion phases, resulting in the selection of 122 peer-reviewed articles relevant to digital twins in precision dairy nutrition.

An initial pool of 300+ papers was retrieved, which was then narrowed down to 280 after removing duplicates. In the second stage, titles and abstracts were read to assess whether the work addressed digital twin systems in a technical or application-driven manner. This left a refined set of 200 candidate papers. The third phase involved a detailed full-text analysis to determine fit with the review’s scope. To ensure methodological rigor and relevance to the scope of digital twin systems in precision livestock nutrition, a set of inclusion and exclusion criteria was applied during the full-text screening stage. Studies were included if they provided a clear focus on digital twin applications in livestock or dairy systems and contributed meaningfully to one or more dimensions of modeling, computational architecture, or implementation. Only peer-reviewed papers that offered sufficient technical detail were retained. Papers were retained if they provided insight into modeling rumination and feeding patterns, data integration architectures, metabolic simulation frameworks, or real-time decision processes in livestock environments. Conversely, papers were excluded if they used the term “digital twin” as a generic label without describing bi-directional data exchange, synchronization, or system feedback mechanisms. During this, we observed some inconsistencies in the use of the term “digital twin”, “digital shadow”, and “cyber physical systems”. In line with Negri et al. [8] and Kritzinger et al. [7], we retained only those studies that demonstrated a bidirectional exchange between physical and virtual systems, real-time synchronization, or feedback-based adaptation. This ensured that the papers met the stricter definition of a true digital twin, excluding papers that used “DT” as a catchphrase. Additionally, articles focused exclusively on crop systems, industrial domains, or unrelated cyber-physical systems were omitted, as were studies lacking methodological transparency or system-level specificity. In the final phase, 122 papers were included for structured analysis. These were organized thematically according to the classification framework in Section 2.1. Figure 4 shows the PRISMA-based flowchart summarizing this screening and selection process, including detailed exclusion criteria.

This methodological approach balances breadth with technical specificity, offering a rigorous foundation for evaluating the architectural, computational, and deployment challenges in designing digital twins for precision dairy nutrition.

### 2.3. Overview of the Data Sources

The studies surveyed in this review draw from a diverse array of data sources, both real-world and simulated, reflecting the interdisciplinary nature of digital twin systems. Within agriculture, most of the diary-focused research relies on sensor-driven datasets collected either on commercial farms or controlled experimental platforms. These include motion data from accelerometers, rumination logs from bolus or collar sensors, ambient temperature and humidity readings, and synchronized feeding events recorded through IoT-enabled feeders and milking systems.

Public datasets like the MMCows [18] provide annotated time-series logs from accelerometer devices, while platforms such as SmartCow (Zenodo) offer multimodal sensor streams combining rumen temperature, motion, and feed intake metrics. Several high-impact studies like “IUMENTA” [19] and a paper on feeding behavior DTs [20] used a mix of real-farm telemetry and synthetic data modeled through Bluetooth or LoRaWAN-based edge infrastructure to simulate biologically accurate digital twins.

Beyond dairy, broader agri-digital twin systems such as AgriLoRa [21] use telemetry collected from GPS-tagged tractors, irrigation control systems, and weather stations. These typically operate at minute-to-hour resolutions and are often integrated with environmental data such as soil moisture or temperature gradients.

The data formats across the reviewed studies are heterogeneous, including structured CSV/JSON time-series, SQL relational logs for health or production cycles, and unstructured data like thermal images or video feeds in animal behavior modeling. Advanced systems like IUMENTA- [19] or FIWARE-based twins [22] use semantic models to represent relationships between farm components such as animals, devices, and environmental variables.

Data frequencies vary by domain. Dairy DTs tend to operate on hourly or daily cycles tied to feeding, rumination, and milking. Manufacturing twins typically log at sub-second intervals while environmental or health-related twins may operate episodically or asynchronously. Several studies have emphasized preprocessing requirements such as temporal alignment, sensor fusion, and filtering for noise. For instance, the Feeding Behavior DT [20] project employed Kalman smoothing to clean accelerometer data, while others used interpolation methods to harmonize bolus, thermal, and motion data streams.

Overall, digital twin systems in dairy nutrition must accommodate a wide variety of data formats and resolutions. Their computational pipelines must be designed to support both low-latency decision-making (like in real-time feeding adjustments) and high-resolution historical modeling (nutrition vs. performance trends across lactation cycles). This diversity underscores the architectural flexibility required for deploying digital twins at scale in dairy environments.

## 3. Digital Twin Architecture

### 3.1. Overview of DT Architecture

The foundational architecture of a digital twin (DT) comprises three interdependent layers—data acquisition, virtual modeling, and real-time connectivity. These components serve as the pillars of the digital twin system that allows real-time monitoring, simulation, and optimization of physical entities. In the context of dairy systems, these architectures must be adapted to handle high-frequency, biologically variable, and spatially distributed data sources while ensuring responsiveness and scalability under resource constraints. According to Grieves and Vickers [6], the DT model should be grounded in a closed-loop feedback structure where the digital model mirrors the physical asset and responds dynamically to changes in its state.

Tao et al. [23] and Wu et al. [10] proposed that digital twins evolve through five layers: the physical layer, where data is collected via sensors and actuators, the communication layer, which is responsible for transmitting this data via LoRaWAN, Bluetooth, or Wi-Fi, the data infrastructure layer, where raw data is filtered, integrated, and stored, the modeling layer, which performs the simulation, prediction, and inferences, and, finally, the decision or application layer, where insights are converted into actions such as feed ration adjustments, health alerts, or farm level reports.

In industrial applications, DTs often rely on centralized cloud-based platforms. However, in agricultural and dairy contexts, hybrid edge–cloud architectures are increasingly favored due to constraints in connectivity, latency sensitivity (for applications like health monitoring), and the need for processing the data in real time and on site. Dairy specific DTs typically delegate simple classification and preprocessing tasks to on-farm edge devices like embedded microcontrollers or Raspberry Pi boards while offloading more complex metabolic simulations or optimization routines to cloud infrastructure.

**Figure 5:**
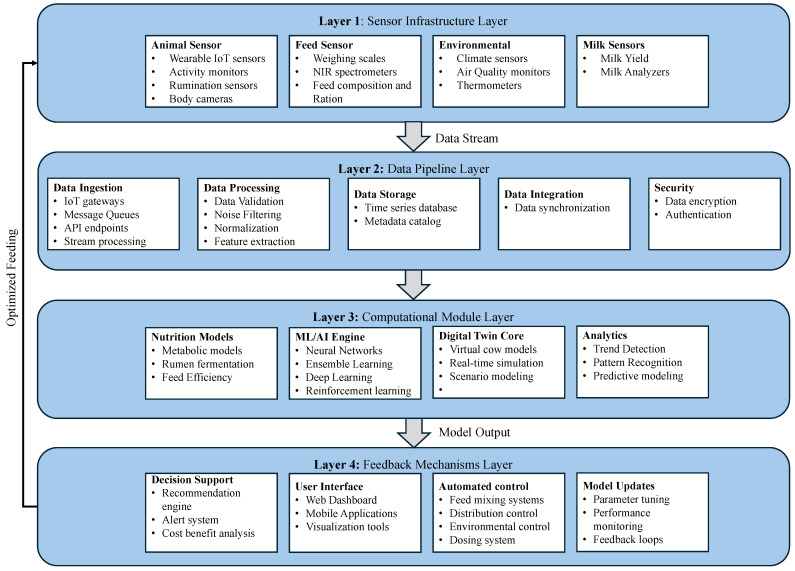
Layered computational architecture of a digital twin system specifically designed for precision dairy nutrition. This detailed schematic illustrates the sequential integration of four operational layers: (1) multimodal sensor infrastructure collecting animal, environmental, and feed data; (2) data pipeline responsible for secure ingestion, preprocessing, storage, and fusion; (3) computational module performing real-time modeling, metabolic simulations, and predictive analytics; and (4) feedback mechanisms delivering actionable insights and facilitating continuous model refinement to optimize dairy production outcomes.

Figure 5 presents a reference architecture adapted for dairy digital twins, illustrating how data flows from cow-mounted and environmental sensors through edge processing modules, cloud-based inference layers, and finally to user interfaces or automated actuators. This layered approach enables modular deployment, which is essential for accommodating diverse farm sizes and technical capabilities.

These layered frameworks have since been adopted across domains, including manufacturing, urban infrastructure, and agriculture, to enable increasingly autonomous, intelligent, and scalable systems.

Recent implementations, such as Digital Twin as a Service (DTaaS), modularize these layers into plug-and-play services for faster deployment and reuse, especially in simulation-heavy environments like autonomous driving and digital livestock systems. Frameworks like IUMENTA [19] are designed for animal digital twins to exemplify a new generation of DT platforms where each component can be independently configured and scaled across species, environments, and resolution levels.

### 3.2. Components of Dairy Nutrition Digital Twin

A digital twin designed for precision dairy nutrition integrates multiple system components across several layers, each responsible for handling a specific aspect of data acquisition, processing, modeling, or intervention. Figure 5 presents a five-layer reference architecture designed to meet the unique requirements of real-time dairy environments, like multimodal sensor integration, physiological modeling, and feedback mechanisms.

The Sensor Infrastructure Layer captures real-time data from various sources within the physical farm environment. Animal sensors, such as wearable RFID tag collars, rumen boluses, accelerometers, and thermal cameras measure motion, rumination, and physiological responses. Feed sensors such as weighing scales and NIR spectrometers collect data on ration intake. Environmental sensors monitor barn climate, air quality, and thermal conditions. Milk analyzers track milk yield and composition. Together, these inputs provide the raw observational basis for the digital twin’s functioning. Livestock-oriented systems like IUMENTA [19] and dairy behavior recognition frameworks [24] demonstrate effective use of multi-sensor fusion by integrating data from various embedded and environmental devices. In industrial setups like Siemens PLM [4] or AutoDRIVE [25], various sensors monitor motion, temperature, vibration, and energy consumption, feeding data at a millisecond-level resolution.

The Data Pipeline Layer handles the ingestion, preprocessing, and storage of sensor data. Data ingestion components include IoT gateways and message queues that receive data streams from the field. Data processing modules perform noise filtering (e.g., Kalman smoothing), normalization, and feature extraction to prepare the data for modeling. This layer handles data integration tasks such as temporal alignment across sensors and applies encryption and authentication mechanisms to ensure data security and privacy before they are input into modeling layers.

At the core of the system lies the Computational Module Layer, where real-time system behavior is simulated, predicted, or optimized. This layer is composed of four main modules. The Nutrition Models [26,27,28] simulate digestion, fermentation, and feed efficiency using compartmental or mechanistic models. The AI/ML Engine includes neural networks, ensemble methods, and deep learning models trained to predict feeding behavior or detect anomalies. The Digital Twin Core orchestrates real-time simulations of virtual cows, combining inputs from both nutrition models and live sensor data. Finally, an analytics module conducts trend detection, pattern recognition, and predictive modeling for performance benchmarking.

Traditional physics-based models are commonly employed in engineering-focused twins such as smart manufacturing systems [29], where machine behavior is mathematically deterministic [30]. In contrast, agriculture and healthcare applications favor data-driven and hybrid modeling due to biological variability. Techniques include BiLSTM and GANs [31] (COVID-19 twins), reinforcement learning, and neural implicit representations [32] for behavior prediction and optimization. Recently, “Digital Twin-Driven Teat Localization and Shape Identification” [33] proposed using convolutional networks to locate and segment anatomical features in dairy cows, improving the precision of robotic milking systems. Similarly, “Cow Daily Behavior Recognition Based on Multimodal Data” [34] employed thermal imaging and accelerometry to classify behavior states using deep CNNs and attention modules. In swine farming, the “Digital Twin Application: Making a Virtual Pig House” [35] integrated temperature, light, and sound feedback into the behavior simulation loop to enhance comfort-based automation. These developments collectively reinforce the shift toward more adaptive, self-calibrating twin models, particularly in biological systems, where behavior and response are context-dependent.

Hybrid models leverage both first-principles physics and learned behaviors to maximize predictive accuracy while retaining interpretability. “Conceptual Digital Twin Modeling Based on TRIZ Function Model” [10] provided a methodology for innovation-driven modeling, bridging conventional control theory with inventive problem solving. “Digital Twin: Generalization, Characterization and Implementation” [36] emphasized adaptability by enabling component-wise modeling across biological and engineered systems. Such flexibility is essential in environments like dairy farming, where both routine and stochastic events impact outcomes.

Table 1 consolidates representative modeling techniques from both agricultural and cross-domain digital twin systems, summarizing their purpose, algorithmic type, and the domains in which they have been applied.

The Feedback Mechanisms Layer closes the loop by transforming insights into actions. Decision support tools generate recommendations for ration adjustments or health alerts. Interfaces such as dashboards or mobile applications can deliver these insights to farmers and veterinarians. Automated control systems may directly operate feed mixers, climate control systems, or dosing equipment based on model output. Finally, a dedicated module handles feedback loops, ensuring continuous model adaptation through parameter updates and performance monitoring.

Feedback loops in digital twins for dairy nutrition are essential for maintaining real-time coherence between the physical cow and its virtual representation. Various studies [12,37] have illustrated the deployment of real-time data from wearable sensors feeding back into the virtual model, which in turn informs feeding decisions or health alerts. Similarly, the IUMENTA architecture [19] employs a loop where bolus sensor input modulates digestion models to fine-tune nutrient delivery.

This modular breakdown of the architecture ensures that the digital twin can support both short-term decisions like feed adjustments and long-term planning like herd health optimization. Moreover, the layered architecture enables scalability across different dairy farm sizes and infrastructure levels, making it adaptable from research stations to commercial farm deployments.

### 3.3. Computational Requirements of a Dairy Digital Twin System

The deployment of digital twin systems in dairy farming requires careful consideration of computational trade-offs, particularly because of the need for architecture that is capable of handling heterogeneous data streams, supporting real-time inference, and operating under the infrastructural constraints typical of agricultural environments. Unlike industrial digital twins that are deployed in high-connectivity and resource-rich settings, dairy digital twins must address the realities of rural deployment, like variable network connectivity, limited on-site computational power, and the biological variability inherent in livestock systems. This section delineates the computational demands of various digital twin components in terms of memory requirements, update frequency, processing complexity, and deployment topology.

Modules responsible for real-time behavior monitoring, such as rumination detection and feeding activity classification, are generally deployed at the edge due to latency constraints. These models process high-frequency data from the accelerometer and bolus sensors and require lightweight architectures to operate efficiently on embedded systems with limited memory and connectivity. Zhang et al. [20] proposed a behavior recognition framework method using a time-series neural network architecture trained on a single collar-mounted sensor per cow. The system achieved high classification accuracy (above 94%) using 5 s windows and demonstrated effective performance in a lightweight hardware–software configuration, which is also suitable for small scale farms. The authors emphasize the platform’s low cost, ease of deployment, and compatibility with real-time monitoring in real farm environments with limited technological infrastructure, making it relevant for scalable digital twin applications in livestock systems.

Similarly, Han et al. [37] implemented hybrid CNN–LSTM architecture to classify cow behaviors (standing, sitting, lying, or walking) based on time-series data from wearable motion sensors. The model was systematically optimized for key hyperparameters, including the hidden neuron count and number of LSTM layers, to balance predictive accuracy and computational efficiency. Their experimental results validated using real-world sensor data collected from cows in commercial settings and demonstrated that the system could achieve accurate classification while maintaining a low average test loss under a modest model complexity of one hundred twenty-eight hidden units and two LSTM layers. The authors highlighted the model’s suitability for use in IoT-enabled livestock environments and its potential for edge deployment in smart farming scenarios where continuous behavior recognition and low-latency inference are essential. While explicit latency figures were not reported, the work provides a framework for real-time, resource-constrained behavior classification in digital twin systems for cattle management.

Conversely, computationally intensive components such as metabolic process modeling and feed optimization, are typically centralized and executed in cloud environments. These modules require access to historical datasets, integration of external factors such as environmental conditions and inventory data, and the execution of computationally demanding algorithms. For instance, in the IUMENTA [19] framework, the authors implemented a cloud-hosted optimization engine using genetic algorithms that simulate multiple feeding scenarios based on a cow’s energy expenditure. The pipeline architecture supports modular data merging across inputs from multiple channels, model training, and report generation. The paper details the pipeline’s scalability and the system’s ability to operate distributed components both locally and on the cloud. These architectural choices ensure flexibility and extensibility of digital twin models across different animal species and hardware configurations. Tzachor et al. [38] highlighted the role of reinforcement learning agents in agri-food digital twins for optimizing agricultural inputs and decision-making across the agri-food supply chain. Their system-level perspective emphasizes the potential of DTs to simulate crop–environment interactions, reduce greenhouse gas emissions, and manage food waste through AI-enhanced control strategies. While the work focuses more on policy and environmental optimization than on dairy-specific nutritional modeling, its framework provides a conceptual basis integrating decision intelligence into livestock-oriented digital twins.

Zhang et al. [34] described a multimodal behavioral recognition system that integrates thermal imaging and accelerometer data to classify daily activities in dairy cows. Their model pipeline incorporates temporal alignment, Kalman filtering, and feature-level sensor fusion, enabling robust interpretation of complex animal behaviors across varying environmental conditions. The architecture supports efficient deployment for continuous monitoring in livestock environments.

Latency and communication overhead are persistent bottlenecks in digital twin implementations, particularly in rural agricultural settings. Menges et al. [39] addressed these challenges through a thermal imaging-based predictive digital twin framework that leveraged edge devices to perform local inference. This approach enhanced responsiveness and minimized data transfer delays, allowing for real-time alert generation and on-site decision-making.

A critical limitation in many current DT deployments is the computation and communication tradeoff between edge and cloud components, especially in rural farm environments with unreliable connectivity. Tasks such as behavior classification using accelerometer or bolus data often require low latency (under 500 ms), which is difficult to achieve if all data must be streamed to a central server. Heavier computations like feed optimization or metabolic simulations need more processing power and memory than edge devices typically support. For instance, Zhang et al. [20] demonstrated that edge-deployed LSTM models achieved over 94% classification accuracy for feeding behavior but required frequent sampling (5 s windows) and real-time inference, which cloud-based systems could not deliver due to upload delays. Menges et al. [39] reported that offloading image processing to edge devices significantly reduced inference latency compared to cloud-only pipelines. Their thermal imaging-based DT system generated real-time alerts even in bandwidth-constrained farms, underscoring the responsiveness gains of edge deployment. These cases highlight scalability tradeoffs: edge systems improve responsiveness but may restrict complexity, while cloud systems increase accuracy and interpretability at the cost of delays and bandwidth usage.

The computational profiles of these modules differ not only in their function but also in the required frequency and immediacy of updates. Behavior monitoring and health alerts operate on low-latency intervals and demand continuous data ingestion, while optimization and analytics processes such as lactation curve tracking or performance benchmarking typically function on longer cycles, use a cloud-based data warehouse, and can be executed asynchronously via batch processing.

Table 2 presents an overview of the computational requirements across major modules in precision dairy digital twins. These estimates are derived from reported system specifications [24,34,39,40] and technical parameters identified in digital twin pilot implementations for cattle behavior, nutrition modeling, and environmental monitoring. While exact resource usage depends on implementation and hardware variation, the ranges here reflect consensus values from field-deployed systems and benchmarks. Complexity measures follow standard algorithmic profiling based on input size (n), while memory and data rates are based on sensor resolution and sampling frequencies reported in practice.

The heterogeneity of these demands necessitates a modular deployment strategy. Smaller farms may prioritize localized infrastructure with batch synchronization, whereas large-scale operations benefit from distributed edge–loud ecosystems. Designing these systems requires careful alignment of computational efficiency, inference latency, and model interpretability with practical limitations in bandwidth, energy use, and technical capacity. Precision dairy digital twins must function as distributed, modular systems, combining the immediacy of edge inference with the analytical depth of cloud platforms. Successful implementation depends on the coordinated execution of memory-aware modeling, adaptive scheduling mechanisms, and robust data communication protocols tailored to farm-scale environments. Precision dairy digital twins must be engineered not as monolithic platforms but as distributed systems, optimized for real-time responsiveness at the edge and analytical depth in the cloud. Successful deployment hinges on the seamless coordination between memory-aware modeling, context-sensitive scheduling, and adaptive communication protocols.

### 3.4. Communication, Middleware, and Standards

Effective communication infrastructure and middleware form the operational spine of digital twin ecosystems, enabling seamless data exchange between physical systems, edge analytics modules, and centralized computational services. In dairy-specific digital twins, these requirements are heightened by the need to maintain low-latency synchronization across heterogeneous devices deployed in bandwidth-limited rural environments.

Middleware protocols such as MQTT (Message Queuing Telemetry Transport), OPC-UA (Open Platform Communications—Unified Architecture), and DDS (Data Distribution Service) are commonly used due to their lightweight footprint, publish–subscribe architecture, and support for asynchronous updates. These frameworks facilitate real-time telemetry integration from wearable livestock sensors, milking robots as well as environmental monitors into central or edge-based models. In the context of smart agriculture, systems such as AgriLoRa [21] and SmartCow demonstrate the use of LoRaWAN-based communication for connecting distributed sensor networks across vast pastures, while urban-scale digital twins like Herrenberg rely on 5G and NB-IoT networks to coordinate higher-bandwidth modalities like video and LiDAR feeds.

From a software abstraction standpoint, communication middleware is often coupled with semantic integration frameworks, including FIWARE and IUMENTA [19], which utilize RDF (Resource Description Framework) and OWL-based ontologies to maintain machine-readable representations of physical farm entities. Despite this progress, the lack of universally adopted open standards and inconsistent data schemas continues to limit interoperability and scalability across platforms and domains.

A comparative evaluation by Kritzinger et al. [7] and the technical review “Connecting the Twins” [43] explicitly benchmarked communication stacks based on latency, jitter, and bandwidth trade-offs, affirming the trade-off between decentralized responsiveness and centralized robustness. As dairy DTs evolve towards real-time behavioral prediction and metabolic decision support, efficient, fault-tolerant communication architectures will be critical for maintaining continuous operation in the face of rural connectivity challenges.

### 3.5. Integration with AI, Cloud, Edge and Big Data

Modern digital twin implementations are hybrid systems that combine the distributed strengths of edge computing, cloud-based analytics, and AI-driven reasoning models. Particularly in dairy environments, this architecture enables computational segmentation by placing time-sensitive behavioral recognition tasks at the edge (rumination classification) and offloading the heavier population-level optimization and historical trend analysis to the cloud.

AI integration is reshaping digital twin intelligence. Studies by Zhang et al. [20] and Han et al. [37] have shown how LSTM, CNNs, and hybrid CNN–LSTM models can perform real-time behavioral analysis from raw accelerometer data with over 85% accuracy. These models are deployed on edge devices with relatively less memory and low inference latency, enabling responsive decision-making in isolated farm environments.

At the cloud layer, genetic algorithms and linear programming engines are used to solve multi-objective optimization problems, such as balancing feed composition against cost, availability, and animal metabolism. In more experimental systems like Real2Sim2Real [44] and AutoDRIVE [25], the bidirectional transfer of learning from simulation to real-world contexts enables adaptive behavior modeling and autonomous control. This is still nascent in dairy applications but presents promising avenues for cross-modal generalization and digital prototyping.

The computational convergence of AI, edge analytics, and cloud services is enabling closed-loop feedback architectures in digital twins and is linking sensing, reasoning, actuation, and learning in an adaptive cycle.

### 3.6. Strengths and Limitations of Current DT Architectures

Digital twin architectures offer compelling advantages for precision livestock farming, particularly through their modular, layered design. This architecture allows different subsystems such as behavior analysis, environmental sensing, and feed optimization to operate asynchronously yet cooperatively. This modularity supports incremental deployment in farms with varying levels of technological readiness.

However, critical limitations persist. Synchronization between various asynchronous data sources such as bolus sensors, accelerometers, and feed intake logs remains technically complex, especially in the absence of standardized timestamps or sampling frequencies. Furthermore, many commercial twin frameworks operate as closed ecosystems, restricting interoperability and increasing vendor lock-in.

From a computational perspective, high-frequency models such as those used for behavior recognition impose energy and memory constraints when deployed at the edge, while cloud-based optimization engines can suffer from delayed responsiveness due to connectivity gaps in rural infrastructure. Balancing these constraints remains a core architectural challenge and requires context-aware decisions about model placement, data flow, and latency tolerance in deployments. The interpretability of AI models, especially deep neural networks, is also a concern. Current DT deployments often act as “black boxes”, which reduces trust among veterinarians, farm managers, and regulators.

Addressing these challenges will require the use of open, interpretable architectures such as Explainable AI. It will require edge-first deployment strategies and federated learning protocols to minimize data transfer latency while retaining predictive accuracy. Emerging frameworks like IUMENTA [19] advocate for open, standard-based interfaces with reusable ontologies to encourage ecosystem-wide integration and innovation.

## 4. Computational Methods in Precision Dairy Digital Twins

The implementation of digital twins (DTs) in precision dairy nutrition requires multidisciplinary integration of biological models, data analytics, and system-level computing and hence relies heavily on a diverse set of computational techniques. These methods span real-time behavior recognition, metabolic modeling, optimization of nutritional inputs, and scalable edge–loud analytics frameworks. This section synthesizes the algorithmic and system-level approaches used in contemporary research, focusing specifically on their utility and application within the dairy sector.

### 4.1. Rumination and Feeding Behavior Recognition

Accurate behavioral modeling in dairy cows is a critical first step in constructing an accurate digital representation. Time-series classification techniques, especially those leveraging deep learning architectures like CNNs and LSTM, are commonly used for detecting rumination, eating, and resting behaviors from accelerometer and bolus data.

Zhang et al. [20] proposed a lightweight CNN–LSTM hybrid model trained on multivariate sensor streams to classify eight core behaviors in dairy cows from tri-axial accelerometer and bolus sensor data. Their architecture utilizes 5 s sliding-window intervals and is optimized for real-time inference on resource-constrained edge devices, achieving accuracies over 90%. The study highlights the model’s edge suitability through low inference times, low memory utilization, and low costs.

Han et al. [37] extended this by using LSTM models for multi-behavior classification using wearable sensors, with a focus on latency-aware design architecture. This implementation supports real-time updates from the custom collar around the cows’ neck, allowing dynamic state transitions to be captured without centralized cloud computation. These techniques often require preprocessing steps such as Kalman filtering [45], signal smoothing, and feature extraction, which are essential for modeling behavioral state transitions that inform nutritional demand.

### 4.2. Metabolic Modeling Techniques

A core component of digital twin frameworks for dairy applications lies in the modeling of metabolic processes. It acts as the nutritional feedback loop in a DT by simulating internal physiological states based on sensor input and historical patterns. They simulate feed intake, rumen fermentation, and nutrient partitioning in dairy cattle. Physics-based models employing compartmental dynamics and differential equations are widely used. They reflect the structured dynamic flow of substances through the digestive and metabolic pathways.

McNamara [46] provided a foundational review of ruminant systems modeling using compartmental differential equations, highlighting their predictive utility and biological fidelity. These models divide the ruminant body into subsystems, integrating nutrient kinetics over time. The simulations can be executed iteratively in farms using inputs like feed intake, pH, rumen temperature, and milk yield to maintain up-to-date metabolic states. The author also noted their utility not only in academic modeling but also in precision nutrition applications when combined with dynamic data and on-site computing capacity. Similarly, Johnson et al. [47] demonstrated early use of dynamic simulation to teach systemic metabolic control, which has since been adapted into digital twin contexts. These simulations are now updated hourly using live data inputs, such as ruminal pH, feed composition, temperature, and milk yield, and executed on farm-side GPUs to enable near-real-time updates.

Studies by Kebreab et al. [48,49] and Muñoz-Tamayo et al. [41] further advanced metabolic modeling using mass balance frameworks and fermentation system dynamics. In particular, Muñoz proposed a multi-compartmental rumen fermentation model that simulates substrate degradation, microbial dynamics, and volatile fatty acid (VFA) production based on in vitro experimental validation. This model had been referenced as a strong candidate for real time digital twin integration because of its mechanistic fidelity and adaptability to sensor inputs.

Further, González et al. [50] proposed a Bayesian calibration approach for parameter estimation in metabolic models of ruminants, specifically to manage the biological variability between different cows. This method enables real-time updating of model parameters using streaming data such as ruminal pH, body temperature, and milk components, which are key for adaptive digital twin operations.

Lastly, from an implementation perspective, these models are computationally expensive. Real-time implementation of these models typically occurs on local servers or edge devices equipped with GPUs, as seen in Zhang et al.’s study [34], where thermal imaging and metabolic inference were fused to derive individualized twins that mirrored behaviors.

### 4.3. Optimization Algorithms for Feed Formulation

Once behavioral and metabolic states are inferred, optimization algorithms guide the formulation of feed rations, giving actionable feeding strategies. These algorithms are tasked with formulating feed rations that not only satisfy nutritional needs but also account for operational costs, resource availability, and environmental goals such as methane mitigation. Among the most widely adopted techniques are linear programming (LP), dynamic programming, and genetic algorithms (GAs).

Linear programming has a long-standing role in livestock nutrition optimization. It enables cost minimization under a set of nutrient constraints, relying on predefined feed composition values and predicted animal requirements. These systems encode nutritional requirements as inequality constraints and solve for cost-minimizing feed blends using updated coefficients observed from the digital twin. Youseff et al. [19] describe a feed management subsystem where updated feed models are automatically triggered by behavior classification outputs and physiological indicators like reduced chewing duration or anomalous rumen temperature fluctuations.

Where linear models struggle with non-linear interactions and incomplete information, metaheuristic methods like genetic algorithms (GAs) offer a flexible alternative. They can handle more complex and non-linear optimization challenges. Atıcı and Elen [40] developed a GA-based system that minimized feed cost while adhering to nutritional constraints such as metabolizable energy, crude protein, fiber fractions (ADF, NDF), calcium, and phosphorus. Their model selected from a library of feed components and demonstrated the GA’s ability to effectively navigate large combinatorial spaces. GAs are particularly suitable for digital twins requiring flexibility in objective functions and variable constraints, such as those factoring in methane emissions or feed availability. This adaptability makes them especially useful in cloud-based digital twin architectures designed for multi-farm or cooperative networks.

Recent advances also explore hybrid approaches that couple reinforcement learning (RL) with classical solvers. These agents can simulate the long-term metabolic and economic consequences of various feeding strategies in silico before applying them to real animals. Such pipelines are under exploration in several academic prototypes and early commercial deployments, though publicly available documentation remains limited.

These optimization engines typically operate on daily or on-demand cycles, balancing the need for up-to-date nutrition decisions with computational overhead and latency considerations. In edge-deployed scenarios, lightweight LP solvers are favored for near-real-time updates, while more computationally intensive GA-based systems are run centrally and synchronized periodically.

In summary, optimization modules in digital twins are evolving from static calculators to intelligent, self-adaptive systems. They serve as the decision-making core of precision nutrition pipelines, integrating continuous sensor feedback, behavioral forecasts, and metabolic simulations to guide dietary interventions with increasing autonomy and precision.

### 4.4. Edge Computing and Real-Time Infrastructure

Digital twins for precision dairy nutrition must operate in environments with stringent latency, bandwidth, and energy constraints. These limitations make edge computing essential for ensuring timely model inference, robust system responsiveness, and reduced dependency on unreliable rural connectivity.

A representative implementation was described in the thermal imaging-based DT system by Menges et al. [39], where local edge devices were used for on-site image processing and behavioral inference. Their architecture was designed specifically for livestock monitoring and reported a 70% latency reduction compared to traditional cloud-based pipelines. The reduction was primarily achieved by deploying CNN-based detection models directly on embedded GPUs located within the barn infrastructure, enabling continuous health status monitoring without requiring full video upload to the cloud. In addition, data orchestration between devices is handled using robust event streaming frameworks. Technologies such as Apache Kafka (v2.0+) and Apache Spark (v2.4+) support high-throughput, fault-tolerant message passing and batch analytics. These are often deployed alongside lightweight containerization platforms like Docker, which facilitate modular service deployment across heterogeneous hardware environments ranging from NVIDIA Jetson modules at the edge to centralized data lakes in cloud clusters.

Shen et al. [51] developed an edge-based system capable of recognizing cow ruminating behavior in real time using a custom edge device equipped with a three-axis accelerometer and STM32 microcontroller. This system processed behavioral data locally and only transmitted summarized results to the cloud every two hours, reducing network traffic by 99.9% compared to raw data uploads and achieving an accuracy of 96.1% for behavior classification, substantially improving responsiveness and energy efficiency in continuous health monitoring scenarios. Complementing this, Alonso et al. [52] introduced the SmartDairyTracer platform based on the Global Edge Computing Architecture (GECA), which integrates IoT, edge, and blockchain technologies in a modular, multi-layered setup. Their system demonstrated effective real-time monitoring of both livestock and feed grain, enhanced data traceability, and reduced latency through edge–local AI-driven analytics on mixed dairy farms in Spain.

Collectively, these systems illustrate a paradigm shift from cloud-centric to edge-first strategies in digital twins. The cumulative advantage of edge computing lies not only in reduced latency but also in enhanced system autonomy and fault tolerance. By offloading the bulk of computation to on-site infrastructure, farms become less dependent on external servers and gain the ability to sustain critical operations during cloud service outages or network instability. Table 3 summarizes this shift by contrasting edge-only, cloud-only and hybrid models across latency, accuracy, cost, and scalability dimensions.

### 4.5. System Integration and Middleware Design

Effective deployment of DTs depends on seamless integration with farm management systems and bridge heterogeneous data sources, legacy infrastructure, and modern analytics platforms. Middleware architecture plays a critical role in bridging these technical layers.

FIWARE-based frameworks and IUMENTA provide API-first designs that ensure compatibility across devices and vendors. These platforms support modular extension, where new devices or subsystems such as milking robots or feeding control units can be integrated without major reconfiguration like a plug and play model. The IUMENTA framework provides a semantic layer that supports data standardization and knowledge modeling for multi-domain interoperability when combining behavioral, nutritional, and health data streams.

Communication protocols like MQTT [53] and OPC-UA support asynchronous messaging between modules. These allow system components to operate semi-independently and maintain functionality during network disruptions. This middleware is often bundled with lightweight brokers like ioBroker and Node-RED to manage real-time control and dashboard visualization with minimal computational overhead.

The ASABE study by Treiber et al. [54] presents a compelling case for middleware-driven integration using a digital broker model implemented at the TUM dairy research farm. Their setup integrates a wide array of wireless sensor networks (WSNs), actuator systems, and external farm management systems (FMISs) into a single interface. This model utilizes Raspberry Pi-based local brokers to unify data from multiple proprietary systems via API connectors, and it supports real-time control, historical storage, and dashboard visualization through Node-RED and custom UI layers.

Connectivity and interoperability remain challenges, especially in rural or resource-constrained regions. To address synchronization issues under intermittent connectivity, middleware must support fault-tolerant communication. Lightweight messaging protocols such as AMQP and MQTT ensure queued delivery and retransmission of missed packets, while semantic representations using RDF/OWL enable data contextualization across modules. These ontologies define inter-variable relationships and power rule-based alert systems and predictive models.

Security architecture for digital twins in precision livestock farming must align with both information security best practices and the unique constraints of operational technology [55]. Authentication and encryption standards such as TLS 1.3, secure device provisioning via X.509 certificates, and role-based access control are becoming standard within platforms like Azure IoT and AWS Greengrass. These measures ensure end-to-end integrity and confidentiality, particularly in systems transmitting biometric and health data [56].

Legacy system integration presents another major technical challenge. Many farms still rely on proprietary herd management software or siloed data storage solutions. Middleware bridges such as OPC-UA gateways or schema translation modules have been proposed to convert legacy data into standardized ontologies like SAREF-Agri or SSN/SOSA. These can be implemented via ETL (Extract, Transform, Load) processes that run in background intervals, ensuring backward compatibility while enabling integration into modern DT pipelines.

To understand the computational methods used in precision dairy nutrition, we compare various modeling approaches in Table 4. These include physics-based models, machine learning models, hybrid approaches, and agent-based simulations. Each approach has its strengths and weaknesses depending on the application, data requirements, and computational efficiency. While hybrid models integrate elements from both mechanistic and data-driven methods, we present them here as a comparative modeling approach within the broader modeling paradigm. This comparison highlights how different models can be leveraged across varying use cases. Together with appropriate middleware strategies, these modeling approaches ensure that the digital twin ecosystems remain resilient, secure, and extensible across varying farm sizes, infrastructure maturity levels, and evolving hardware landscapes.

### 4.6. Advanced AI Enabled Modeling Paradigms in Digital Twin Systems

As digital twin (DT) systems evolve from conceptual frameworks to operational tools for precision dairy nutrition, artificial intelligence (AI) is redefining how these systems model, interpret, and optimize biological complexity. This section highlights three frontier areas—foundation models, physics-informed machine learning, and advanced multi-objective optimization—that are transforming the computational foundations of livestock digital twins.

#### 4.6.1. Foundation Models and Large Language Models in Livestock Health Data Integration

Foundation models, particularly large language models (LLMs), have introduced a new paradigm in agricultural data interpretation. Trained on vast corpora and adaptable with minimal fine tuning, LLMs offer significant advantages over traditional rule-based systems. In dairy-focused digital twins, they enable the integration of unstructured data such as veterinary records, feeding logs, and farmer observations, which were previously difficult to incorporate into analytical models [60,61].

Boguslav et al. [62] demonstrated the power of these models by fine tuning multiple clinical LLMs on 246,000 veterinary electronic health records. The resulting systems accurately assigned SNOMED CT diagnostic codes across 7739 categories, outperforming prior methods such as VetTag. Notably, even LLMs with modest computational resources produced robust outputs, indicating their suitability for farm-level deployments. Sivakumar and Neethirajan [63] further expanded this potential by integrating LLMs with multimodal data streams, including video, audio, and sensor input. Their work showed that LLMs could support early anomaly detection by correlating behavioral data with subjective human observations, strengthening the twin’s ability to infer real-time health states. In addition to interpretation, LLMs are now being used for decision support. Techniques such as retrieval-augmented generation (RAG) allow these models to synthesize information from the scientific literature and generate nutritional recommendations based on farm-specific context [61]. This dynamic capability surpasses traditional expert systems by providing continuously updated, evidence-based advice.

#### 4.6.2. Physics-Informed Machine Learning for Metabolic Modeling

Physics-informed machine learning (PIML) represents an important advancement over purely data-driven methods by embedding biophysical constraints directly into neural networks [64,65]. In dairy systems, these models enable accurate simulation of processes like rumen fermentation, nutrient partitioning, and manure management while maintaining consistency with established biological principles [66,67]. Genedy et al. [66] applied a physics informed neural network (PINN) to predict manure temperature during storage and found that it significantly outperformed conventional neural networks and finite element models. Their PINN achieved R-squared values above 0.70 during testing, compared to as low as −0.03 in traditional approaches. These results underscore the benefit of incorporating physical constraints in agricultural simulations.

The strength of PIML lies in its ability to encode conservation laws and system dynamics. For example, in rumen modeling, equations governing mass balance, microbial metabolism, and pH buffering can be embedded into the model’s structure [68]. This ensures that predictions remain biologically plausible even under previously unseen conditions. More recent innovations such as Physics-Informed Kolmogorov Arnold Networks (PIKANs) have improved model optimization and introduced uncertainty quantification [64]. These frameworks can simulate diverse physiological interactions including the Fick Henderson equation for pH dynamics, nutrient absorption models, and energy balance formulations relevant to milk production [67]. This fusion of domain knowledge and machine learning provides interpretable and scalable tools for real-time digital twin deployments.

#### 4.6.3. Advanced Multi-Objective Optimization Using NSGA III

The complexity of modern dairy nutrition demands optimization algorithms that can balance numerous objectives including cost, emissions, health, and productivity. The Non-Dominated Sorting Genetic Algorithm III (NSGA III) addresses this challenge by navigating many objective spaces that exceed the limitations of classical solvers [69,70,71]. NSGA III operates by evolving a population of solutions that represent different nutritional strategies. It uses reference point-based selection and non-dominated sorting to identify Pareto-optimal combinations of inputs such as feed ingredients, supplement levels, and feeding schedules [72]. The result is a diverse solution set that allows farm managers to visualize trade offs and select strategies aligned with their goals.

Dang et al. [71] demonstrated the effectiveness of NSGA III in a precision fertilizer system, optimizing simultaneously for cost, accuracy, and environmental safety. In dairy systems, the same principles apply to optimizing nutritional formulations under multiple, often conflicting constraints. The algorithm has also been deployed successfully in crop modeling for balancing yield, resource use, and sustainability [73,74]. For dairy digital twins, NSGA III enables dynamic adjustment of diets in response to real-time changes in lactation stage, environmental stress, or feed availability. Improvements in parallelization and hybridization with other solvers have enhanced the speed and scalability of NSGA III, making it viable for large herds with individualized nutritional requirements [75].

#### 4.6.4. Integration and Synergistic Applications

The most impactful digital twin systems will combine the strengths of foundation models, physics-informed learning, and advanced optimization. For example, LLMs can extract health conditions from veterinary records, while physics-informed models simulate the cow’s metabolic response to feeding changes. These outputs can then inform NSGA III algorithms that identify optimal nutritional strategies across cost, performance, and welfare metrics [76]. This kind of integrated workflow enables personalized decision-making that is biologically grounded, computationally efficient, and context-sensitive. It also opens the door to adaptive digital twins that evolve with each animal, supporting proactive interventions based on real-time feedback from farm conditions. The convergence of these AI techniques defines the next generation of precision livestock management. It enables digital twins not just to reflect the physical world but to understand, predict, and optimize it in ways that are interpretable, reliable, and scalable.

### 4.7. Comparative Assessment of Tranditional vs. DT-Based Livestock Management

Traditional livestock management has long relied on manual practices such as visual estrus detection, periodic weighing, and fixed feeding schedules. While these methods are cost-effective in low-tech environments, they often suffer from limitations in accuracy, labor efficiency, and scalability. In contrast, DT systems offer real-time data integration, behavior modeling, and automated decision-making, thereby enabling more personalized and adaptive interventions.

In nutrition, traditional ration balancing often depends on generalized NRC-based thumb rules or manual feed adjustments. These approaches do not account for dynamic changes in individual animal needs or environmental stressors. DT-based systems leverage behavioral data to dynamically update nutrient models, enabling adaptive feeding strategies [12].

From a cost perspective, while DT technologies incur higher upfront investment, they yield long-term benefits through improved productivity, lower disease incidence, and reduced labor costs. Additionally, field applicability continues to grow with the miniaturization of sensors, the rise of tinyML on edge devices, and increasing connectivity in rural regions [77].

## 5. Digital Twin Integration Framework for Farm Management System

The successful deployment of digital twin (DT) systems in precision dairy farming requires more than modeling accuracy. It relies on robust integration with heterogeneous on-farm systems, modular architecture, and staged implementation. This section outlines a comprehensive integration framework while embedding a phased implementation roadmap, aligned with current field-tested practices.

Modern DT platforms for dairy are increasingly adopting modular API-first designs that support seamless interoperability with farm management information systems (FMISs), IoT sensor networks, and cloud analytics platforms. These ensure that services can interact dynamically across different layers of DT architecture, even under variable network conditions.

Phase 1: Infrastructure and Interoperability Setup

Initial implementation focuses on establishing foundational connectivity. Lightweight brokers such as ioBroker or Node-RED, deployed on devices like Raspberry Pi, allow for local integration of milking units, collar-based sensors, and feed controllers into a unified middleware layer. Protocols like MQTT and OPC-UA provide fault-tolerant communication, buffering messages during disconnections and ensuring reliable delivery, a critical capability in low-connectivity rural areas.

Phase 2: Semantic Modeling and Synchronization

Once data pipelines are operational, the system must support meaningful data integration. This is accomplished through ontology-based data modeling using RDF/OWL standards. Ontologies like SAREF-Agri and SSN/SOSA offer a formal schema to encode domain knowledge, enabling multi-domain interoperability across behavior, health, and nutrition monitoring systems. These semantic layers empower rule-based alerts and automated decision-making, allowing the DT to reason about contextual changes in animal physiology or environment.

Phase 3: Edge–Cloud Optimization and Data Governance

Edge computing strategies are then introduced to reduce latency and bandwidth usage. Case studies such as Menges et al. [39] demonstrate how edge-based thermal imaging DTs achieve over 70% latency reductions compared to cloud-centric models by executing inferences locally. At this stage, data governance policies must also be defined, ensuring farm data sovereignty, secure access (TLS 1.3, X.509), and privacy-preserving analytics via federated learning [78].

Phase 4: Feedback Loop and Farm-Centric Customization

The final stage involves creating a bi-directional feedback system where DT insights drive actionable interventions, personalized feed allocation, early disease alerts, or labor reallocation. Middleware like OPC-UA serves as a bridge for legacy herd management software, enabling ETL processes that convert proprietary formats into standardized digital twin schemas without disrupting existing workflows.

Overall, the ideal system architecture for DT-enabled dairy farms is hybrid: combining loosely coupled services at the edge for time-sensitive operations, with centralized cloud-based orchestration [79] for long-term analytics and cross-farm coordination. Resilience, scalability, and interoperability are achieved through containerized deployment of APIs, semantic data modeling, and adaptive middleware that can negotiate synchronization, security, and translation across diverse farm assets.

## 6. Validation Methodologies for Precision Dairy Nutrition Digital Twins

Robust validation is critical for ensuring the reliability, safety, and generalizability of digital twins in precision dairy nutrition. Unlike static modeling efforts, DTs operate continuously with real-time data streams, requiring multidimensional evaluation protocols that assess both predictive performance and operational resilience. Validation frameworks must be tailored to both the temporal complexity and physiological nuances of livestock nutrition.

Recent studies have emphasized the importance of both offline and online validation methods to ensure the fidelity of digital twin models throughout their lifecycle. Traditional statistical validation methods fall short in capturing transient or localized mismatches between digital representations and real-world dairy system behaviors. Consequently, hybrid approaches that combine data-driven inference, simulation realism, and dynamic trace comparison have gained traction.

### 6.1. Benchmark Datasets and Performance Metrics

Effective DT validation begins with access to standardized datasets representative of real-world farm variability. Public datasets such as the SmartCow project and the ISAEW 2021 proceedings data archive provide labeled behavior and physiological data for cows across regions and seasons. These datasets include synchronized rumen pH, feed intake, milk yield, and accelerometer data, offering suitable inputs for evaluating feeding behavior recognition and metabolic modeling modules. Metrics must be chosen based on module function. Behavior detection models [34] are validated using F1-score, precision–recall, and confusion matrices across behavior classes like rumination, feeding, and lying. Metabolic simulators are validated using root mean square error (RMSE), normalized mean bias (NMB). and Pearson correlation coefficients comparing simulated vs. measured milk yield, nitrogen balance, or methane production [41,48]. Feed optimization engines using linear programming or genetic algorithms are evaluated using cost-efficiency ratios, nutrient compliance scores, and predicted vs. actual productivity response curves.

Lugaresi et al. [80] proposed validation using inter-departure time sequences and material flow traces in manufacturing systems. This approach can be adapted to dairy settings by substituting KPIs with nutrition-specific outputs like milk protein or fat yield and rumen fermentation profiles. Measuring similarity between these real and simulated time-series enables the detection of anomalies or model drift even with limited data availability.

### 6.2. Comparative Evalaution Protocols

Validation of dairy DTs should include ablation studies to isolate the contribution of individual subsystems (e.g., removing pH input from the rumen model or disabling accelerometer-based behavior detection). Comparative baselines can be drawn from rule-based or empirical models, allowing digital twins to be benchmarked against simpler but widely used alternatives. They can also be assessed using sequence-based similarity indicators such as Levenshtein [81] or dynamic time warping distances between real-world dairy performance and simulated outputs. As advocated by Lugaresi et al. [80], validation should distinguish between input accuracy (e.g., ration composition) and model logic fidelity (e.g., digestion kinetics, nutrient partitioning models) to locate the source of discrepancies effectively.

Additionally, cross-platform benchmarking is recommended where cloud-based vs. edge-based deployments are compared under identical data streams, evaluating not only accuracy but latency, data throughput, and inference power draw [39,59,82].

### 6.3. Cross-Validation with Biological Variability

In dairy systems, physiological variation due to lactation stage, parity, or genotype demands validation that generalizes across heterogeneous cohorts. Hua et al. [83] stressed the need for continuous and probabilistic validation frameworks that incorporate real-time IoT-derived data to dynamically update and test model performance. Bayesian calibration and trace-driven simulations allow model parameters to adapt to incoming data while preserving biological realism. Furthermore, they recommend coupling data-driven calibration with logic-based anomaly detection to guard against model overfitting or conceptual drift.

### 6.4. Continuous Model Improvement Frameworks

Digital twins in dairy nutrition are not static artifacts but evolving systems. Lugaresi et al. [80] recommend real-time model updates via online validation that distinguishes short-term deviations from structural errors. For instance, a sudden drop in predicted milk urea levels, uncorrelated with feed nitrogen inputs, may signal model degradation or sensor misalignment. Incorporating sliding window-based validation metrics ensures models maintain predictive accuracy over time.

For ongoing validation and continuous improvement, feedback loops must be implemented using live farm telemetry. This includes monitoring of deviations between predicted and observed values, automatic reweighting of training data, and re-triggering of calibration workflows. In precision nutrition, where feed costs and cow health are tightly coupled, such mechanisms are not only beneficial but essential for ensuring model trustworthiness and avoiding economic losses due to misestimation.

## 7. Applications of Digital Twin

Digital twin (DT) technology has found diverse applications across sectors, ranging from industrial automation to agriculture, healthcare, and urban planning. Digital twins enable continuous monitoring, simulation, and control by integrating real-time sensing with AI driven modeling, visualization, and feedback systems. Additionally, its flexibility enables domain-specific customization. This section critically analyzes the breadth and depth of DT applications by domain, drawing from various published works and highlighting specific technologies, outcomes, and implementation strategies.

### 7.1. Industrial Applications

Manufacturing is the most mature domain for DT implementations, particularly under the umbrella of Industry 4.0 initiatives and cyber-physical integration. The earliest DTs were conceived for machine health diagnostics and virtual commissioning of production lines.

Predictive maintenance and machine optimization were among the first use cases. Studies by Tao et al. [29] and Khajavi et al. [4] demonstrated the use of DTs for predictive maintenance, production optimization, and real-time machine monitoring. Digital twins of industrial lines, such as Siemens PLM [4], support fault diagnosis and asset lifecycle management with remote supervision. The AutoDRIVE ecosystem [25] and digital twin-based virtual commissioning [84] highlight advanced simulation and control strategies integrated with cyber-physical systems. The AutoDRIVE Digital Twin Platform is a fully integrated and extensible cyber-physical testbed designed to simulate autonomous driving in realistic smart-city environments. It has been deployed in field-like educational and experimental scenarios, combining real-time SLAM, V2X communication, multi-agent coordination, and vehicle-in-the-loop (VIL) simulation using RViz. The platform emphasizes reproducibility and hybrid cloud–edge orchestration through ROS-based APIs and containerized modules, validated through 10+ test deployments in autonomous parking, obstacle avoidance, and traffic signal response.

Other implementations, such as the Smart Factory case studies [17,85] documented the integration of SCADA, OPC-UA middleware, and digital replicas for energy optimization and logistics planning. Applications to electric mobility and mechanical systems have also been demonstrated [86].

Most recent DTs are focused on energy efficiency, supply chain coordination, and quality control. “Energy Digital Twins in Smart Manufacturing” [87] used IoT energy meters and real-time analytics to reduce downtime and carbon emissions. “Smart Manufacturing with DT” [88] deployed ML models to detect defects, optimize task sequences, and simulate environmental impact. A study on digital twins for smart grid lifecycle management [89] connected energy infrastructure to broader industrial DTs for fault prediction and load balancing. These industrial examples reflect the transition from static models to autonomous, AI-augmented control systems.

### 7.2. Healthcare Applications

Digital twins in healthcare are less mature but rapidly expanding. Healthcare digital twins are emerging for both personalized medicine and population-level public health. PsyDT [15] is one of the first examples of a LLM-powered digital counselor. It applies large language models to simulate psychological counselors, enabling personalized digital therapy. “Digital Twins in Precision Public Health” by Boulos and Zhang [90] demonstrated how DTs could be used to model the progression of chronic diseases like diabetes or cardiovascular illness by integrated EHRs, genomics, and wearable data. “Digital Twin for COVID-19 Forecasting” [31] predicted infection trajectories using real-world epidemiological data using BiLSTM and GANs. This DT combined a modified SEIRS epidemiological model with Bidirectional LSTM (BiLSTM) and Generative Adversarial Networks (GANs) to simulate and forecast pandemic spread in an idealized UK town. The BiLSTM was trained to learn infection dynamics from real UK virus transmission data, while the GAN generated realistic infection trajectories. These AI-enhanced digital twins provided fast, adaptive, and spatially aware simulations that outperformed static epidemiological models in both prediction accuracy and speed, an approach that could be extended to new pandemics or applied at municipal levels.

Hospital Resource Optimization Twins (Minerva et al. [2]) model hospital-level implementations to optimize bed occupancy, ventilator usage, and emergency routing.

These systems often integrate wearable IoT data with clinical databases to enable dynamic patient-specific simulations. Healthcare DTs often use multimodal data (like EHRs, wearable sensor logs, and NLP outputs), time-series prediction (symptom escalation and medication compliance), and synthetic patient generation for testing clinical workflows or AI models. Challenges include regulatory constraints, interpretability, and patient privacy. “Digital Twins in Healthcare: Theory and Practice” [91] outlined these in detail and proposed solutions involving federated learning and edge AI.

### 7.3. Urban Planning and Smart Cities

Urban digital twins are revolutionizing how cities plan, simulate, and manage infrastructure. The Herrenberg case study [92] utilized real-time citizen feedback loops for smart traffic and mobility systems. The system was rendered in virtual reality and integrated with COVISE for collaborative planning, enabling residents and stakeholders to interact with and modify urban planning scenarios in real time. This participatory model reduced the gap between technical planners and non-experts and enhanced democratic engagement in the town’s 2030 mobility plan.

Sensors embedded in traffic signals, buses, and air quality monitors are fed into a unified control dashboard. Studies from Bogotá [93] and AEC-FM industry reviews [17] illustrate how BIM and GIS integration allows planners to model energy demand under different infrastructure proposals. CitySim [94] used a drone-based vehicle trajectory dataset for vehicle behavior modeling, which is useful for mobility planning and traffic safety policy testing. The project collected over 23 h of video across six diverse urban intersections using UAVs, extracting more than 5 million vehicle trajectories. The digital twin simulates real-time interactions among vehicles, mapping turning patterns, speed distributions, and near-miss events. It supports safety-oriented urban planning, such as evaluating the effectiveness of redesigned intersections or speed calming interventions. CitySim’s architecture integrates edge-based video processing with cloud-based scenario simulations, demonstrating the role of digital twins in enabling proactive, data-driven traffic policy decisions. The dataset and platform are openly accessible, encouraging reproducibility and cross-city comparison.

A review of DTs in the AEC-FM industry [95] discussed the use of DTs in smart building management, predictive maintenance, and energy budgeting. The projects used 3D scanning, BIM, and thermal mapping to create DTs for municipal buildings. Smart grid and transportation integrations [89,96] outlined the interaction between electric vehicle charging and public transit demand. Urban DTs integrate sensor networks (traffic, pollution, energy meters, and noise), citizen feedback portals, and high-resolution 3D modeling using LiDAR.

Limitations in this domain include scaling infrastructure, data governance, and inter-agency interoperability.

### 7.4. Agricultural Applications

Agricultural applications, particularly in livestock management, leverage DTs for behavior analysis, precision feeding, and sustainability tracking. Several emerging works illustrate this evolution toward multi-layered, cyber-physical farm intelligence.

Livestock DTs include systems like IUMENTA [19], which offers a species-agnostic DT framework that simulates physiological status, behavior, and welfare metrics using multimodal senor inputs. A study on smart livestock farms using DTs [12] explored wearables like rumen sensors, accelerometers to monitor feed intake, locomotion, and temperature. Its plugin-based architecture supports cross-device compatibility and promotes generalizability across species, addressing a major scalability bottleneck in livestock DT deployment. A study on feeding behavior DTs [20] used deep learning and edge processing to detect anomalies in chewing and rumination patters, which are crucial for early disease detection. AI-based DTs for cattle caring [37] model each cow’s stress, hydration, and feed efficiency to dynamically adjust rations via smart feeding systems. Digital Twin perception for cattle feeding [34] uses thermal and motion fusion for assessing group behavior under variable climate conditions.

A notable real-world implementation is the Digital Twin Pig House developed by Jeong et al. [35], which virtually replicates a commercial pig facility and synchronizes it with live sensor data to dynamically optimize HVAC systems. By simulating various environmental conditions and actuator responses, the DT reduced energy usage by up to 26.8% while maintaining optimal thermal comfort for the pigs. This system integrates weather forecasts, behavioral patterns, and indoor environmental feedback, and demonstrates how DTs can be used not only for monitoring but also for automated control and predictive operation in livestock infrastructure.

For crop and infrastructure DTs, AgriLoRa [21] integrated LoRaWAN sensors, reinforcement learning, and local climate data to simulate crop and soil health. This supports precision irrigation and fertilizer scheduling. Digital twin-assisted greenhouse irrigation [97] uses soil moisture and weather forecasting to optimize irrigation cycles and reduce water waste. Emerging horticulture-focused DTs extend these methods to root-zone monitoring [98]. “From Bytes to Farm” [99] describes the adaptation of industrial twins to farming applications using open-source telemetry platforms and modular machine learning models.

These case studies confirm that digital twin-supported systems help in reducing methane output, improving feed-to-milk ratios, lowering veterinary interventions, and forecasting yields under climate variability. However, key challenges include connectivity in rural zones, sensor accuracy, and farmer training. Solutions like FIWARE-based twins, semantic APIs [19], and low-power LoRa [21] devices are being tested to address these barriers.

Table 5 presents additional agriculture-focused DT frameworks, demonstrating the diversity of design and scope across livestock and crop systems.

These case studies confirm that industries are moving from traditional data analysis to proactive, twin-driven decision support systems. However, each sector continues to face unique barriers, ranging from rural connectivity in agriculture to data governance in urban planning and regulatory constraints in healthcare. Table 6 presents a comparative overview of digital twin applications across major sectors, outlining their primary functional roles, representative studies, data sources, and implementation challenges. This highlights how each domain tailors digital twin design based on specific infrastructure maturity, sensor types, and operational demands.

## 8. Challenges and Limitations

Despite their transformative potential, digital twin systems face numerous challenges that hinder their widespread adoption and effective deployment. These challenges are both technical and contextual, including issues like data privacy, computational complexity, legacy system integration, and real-time analytics. They range from technical and infrastructural limitations to regulatory, ethical, and cost-related concerns. This section critically assesses these limitations based on an analysis of key papers and real-world case studies.

### 8.1. Data Privacy and Security Concerns

One of the most cited concerns is data security and user privacy, especially in healthcare and livestock DTs, where sensitive biological and behavioral data are involved. The continuous bi-directional data exchange that defines a DT raises substantial privacy risks.

SmartAgriFood and Fractals [22] demonstrated that data governance and ownership disputes are common in cooperative agricultural systems. Farmers are often wary of cloud-based twins, fearing data misuse by equipment vendors or government agencies. PsyDT [15], which models psychological interactions using LLMs, had to rely on generated datasets to avoid violating patient confidentiality. In smart cities like Herrenberg [92], personal mobility traces and audio data from sensors could reveal identities, necessitating differential privacy mechanism.

Cybersecurity attacks on DT infrastructure are also increasing. Digital twins for smart grid lifecycle management [89] highlight how compromised DTs could misreport energy loads or divert critical flows, creating cascading blackouts. “Digital Twins: State of the Art” [29] says that health telemetry streams can be intercepted unless secured with end-to-end encryption and access control protocols. Cybersecurity frameworks are still evolving to match the scale and heterogeneity of DT ecosystems [17], including federated digital twin models, blockchain-secured audit trails, and edge AI for privacy preserving inference.

### 8.2. Real-Time Data Processing and Analysis

DTs depend on the continuous flow of real-time data, yet processing this information at high speeds remains difficult, particularly for complex environments like cities or farms. Latency and bandwidth constraints in edge-to-cloud pipelines [4] hinder real-time feedback. Solutions like edge analytics and federated learning are being tested but require further standardization. Computational demands also grow with data volume, requiring scalable infrastructure and high-performance computing platforms.

AgriLoRa [21] uses LoRaWAN and edge devices to enable low-bandwidth, asynchronous data delivery but even this had limitations during high-throughput or event-based scenarios. Digital twin architecture evaluation for intelligent fish farms [101] showed that data lags caused by rural LTE and underwater sensors impaired the ability of DTs to predict behavior. Papers on digital twins in smart manufacturing [100] highlight the use of Apache Kafka, DDS, and Spark to handle millisecond resolution data, but these require heavy infrastructure investments.

In “From Bytes and Farm” [99], the transferability of industrial DT tools to rural agricultural environments was constrained by the lack of real-time edge gateways. Weather data, cow rumination logs, and greenhouse CO_2_ levels often come with significant temporal misalignment, degrading model performance.

### 8.3. Integration with Legacy Systems

Many industrial and agricultural facilities operate on legacy control systems that were not designed for digital interfacing. Traditional farms, hospitals, and factories were not designed for real-time telemetry, modular APIs, or semantic data models.

“Digital Twin—Proof of Concept” [102] discusses the mismatch between domain-specific operational workflows and cross-domain DT models. Studies like “Digital Twin Architecture Evaluation for Fish Farms” [101] and “Connecting the Twins” [43] reveal frequent incompatibility between DT middleware and old PLCs or SCADA systems.

Bridging this gap requires middleware adapters [19], API translation layers, or complete infrastructure upgrades—each with their own constraints. “Shaping the DT for Design and Production Engineering” [30] recommends ontology-based abstraction layers for legacy data mapping.

### 8.4. High Cost and Complexity

DT implementation demands significant upfront investment in sensors, computing infrastructure, modeling software, and human expertise. This is particularly burdensome for SMEs and small-scale farmers.

Recent studies have estimated the cost of implementing digital twins in agriculture across four main configurations. These figures, while not standardized across geographies, are extrapolated from prototype implementations and academic trial deployments rather than commercial-scale rollouts. Basic IoT systems with wireless sensors can be deployed for USD 1000–USD 4000, making them accessible to small–medium farms [103]. In contrast, cloud-integrated DT platforms with continuous data streaming, cloud analytics, and SaaS support can go above USD 1000 in setup costs. Edge AI hybrid systems, which allow real-time inference on farms, incur higher costs, around USD 20,000, due to advanced hardware requirements. These costs are highly variable and depend on factors such as region, data granularity, and infrastructure maturity. Table 7 summarizes the estimated costs and infrastructure requirements for different digital twin deployment models in precision livestock farming, extrapolated from sources. Modular, open-source deployments can reduce the cost but often at the expense of accuracy and reliability [104].

Papers such as “Digital Twin Technology Challenges and Applications” [105] discuss the economic barriers to adoption. Although no explicit figures are reported, studies such as “From Bytes to Farm” [99] and “AgriLoRa” indicate that the combined expenses of drone based imaging, IoT infrastructure, and predictive analytics in precision farming can exceed USD 20,000 per deployment depending on scale and resolution requirements.

In a study on digital twins in sustainable forestry [106], implementation was delayed due to a lack of funding for permanent sensor towers and real-time meteorological integration. Forest digital twins [107] and smart farming platforms [108,109,110] show that pilot deployments often remain isolated due to cost constraints. Furthermore, AutoRetail Checkout DTs [111] and Product Manufacturing DTs reveal that training ML models for object recognition or assembly fault lines often require thousands of labeled samples, which are expensive to curate.

Simplified DT toolkits and open-source platforms may help mitigate these issues. Promisingly, recent advances in federated learning, low-power edge AI, and open-source model libraries are beginning to reduce setup costs and ease deployment for resource-constrained farms, especially in low- and middle-income countries (LMICs), where traditional infrastructure is limited [78,112].

### 8.5. Model Interpretability and Stakeholder Trust

In sectors like healthcare, agriculture, and governance, decision-making needs to be explainable and transparent. However, ML-driven DTs, especially those using deep learning, often act as black boxes. “Digital Twins in Healthcare: Methodological Challenges and Opportunities” [113] argues that clinics will not adopt opaque models unless they are interpretable, auditable, and regulated. In farming, a study on digital twin perception of cattle behavior [34] noted that while CNNs accurately classified motion states, farmers struggled to understand why a cow was flagged as “stressed”. In smart cities, a study on citizen-centered urban twins [92,93] [Herrenberg, Bogotá] reported resistance from residents when recommendations were unexplained or seemingly biased.

To address these issues, several solutions have been proposed. The Digital Twin for Psychological Counseling (PsyDT) [15] integrates attention-based visualizations to explain language model decisions. IUMENTA [19] provides feedback loops where farmers can annotate system predictions to train more human-aligned models. Researchers propose hybrid twins that combine rules + ML, allowing stakeholders to trace logic chains while preserving adaptability. Recently, Jox et al. [114] proposed a conceptual framework for predictive digital twins in dairy, emphasizing hybrid modeling architectures and explainable AI layers to balance real-time predictive performance with stakeholder interpretability. Their design uses modular logic chains that can be traced and audited by farm operators while remaining adaptive to new sensor inputs.

Beyond interpretability, it is also important to recognize that several architectural choices directly respond to the broader systemic adoption challenges outlined above. While architectural elements like edge computing, middleware, and modular APIs have been mentioned in domain-specific contexts above, they merit cross-domain reflection as deliberate responses to systemic barriers. Edge–cloud balance mitigates real-time data bottlenecks and reduces connectivity demands in rural zones. Middleware frameworks like FIWARE and OPC UA address legacy integration challenges while modular, open-source architectures reduce cost and increase customization flexibility. These strategies highlight a shift from monolithic DT implementations to scalable, layered architectures that align with stakeholder constraints. Rather than being just technical optimizations, such choices enable ethical, affordable, and maintainable deployments across diverse sectors.

In conclusion, digital twin systems face substantial hurdles related to data integrity, real-time synchronization, legacy integration, cost, and trust. However, the ecosystem is maturing. Low-cost sensor packages, edge inference, and open DT frameworks (like FIWARE and IUMENTA) are making adoption easier. Explainable AI, federated learning, and semantic ontologies are closing the gap between automation and human judgment. Addressing these challenges is not optional; it is critical for ethical, scalable, and sustainable deployment of DTs across domains. To consolidate these challenges, Table 8 summarizes the major technical implementation barriers identified in the recent literature, their effects on system performance, existing solutions, and future research directions.

### 8.6. Adoption Barriers Across Farm Scales

While large-scale commercial dairy operations are increasingly piloting advanced DT solutions, smallholder and mid-sized farms face significant barriers to adoption. For example, studies have shown that the high capital costs and limited digital infrastructure often preclude deployment beyond pilot projects [12].

Moreover, platforms like FIWARE [22] and IUMENTA [19] have shown promising results in scalable, open-source DT solutions tailored from LMICs and cooperative farming structures. However, a lack of local technical expertise and support ecosystems limits their real-world scalability.

In the “Farming Reimagined” case study, by Charles et al. [119], autonomous and digitally integrated equipment proved feasible for broadacre farms in Australia, where economies of scale justified high initial investment in RTK-GPS systems, fleet connectivity, and precision actuation.

Conversely, smallholder farmers face structural challenges such as limited capital, weak digital infrastructure, and low digital literacy, which delay or inhibit DT deployment. Customization, affordability, and ease of use emerge as key determinants of success in these contexts, requiring different design priorities compared to commercial farms with advanced machinery and connectivity. Thus, one-size-fits-all DT solutions are unlikely to succeed across the agricultural spectrum.

## 9. Impacts and Benefits of Digital Twin-Based Precision Nutrition in Livestock

The adoption of digital twin technology in precision livestock nutrition holds transformative potential across multiple domains, extending far beyond productivity gains. By facilitating fine-grained behavioral monitoring, individualized nutrition, and predictive health management, DTs offer tangible benefits in reducing greenhouse gas (GHG) emissions, improving resource use efficiency, and enhancing animal welfare.

### 9.1. Enhanced Resource Efficiency

By dynamically adjusting rations based on real-time animal behavior and environmental conditions, DTs minimize overfeeding and feed wastage. Tullo et al. [120] demonstrated that integrating real-time environmental sensors and cow-level data into models enables dynamic adjustment of ventilation and feed delivery, thereby improving feed conversion ratios and reducing energy consumption per unit of milk produced. This precision not only reduces cost but also contributes to more sustainable land and water use.

### 9.2. Greenhouse Gas Emission Reduction

One of the primary sources of emissions in dairy farming is enteric methane, which is tightly coupled with feed intake and digestion efficiency. Traditional feeding systems often result in nutrient oversupply, increasing nitrogen and methane excretion. By enabling the real-time optimization of nutrient delivery based on behavior and physiology, DTs minimize nutrient wastage. For instance, White and Capper [121] demonstrated that diet reformulation based on high-frequency environmental and cow-level data could reduce energy imbalances, thereby lowering the metabolic stress that often leads to inefficient digestion and elevated emissions.

### 9.3. Enhanced Animal Welfare

Real-time behavioral analysis supported by video monitoring and machine learning enables early detection of lameness, stress, or illness. This shifts the model from reactive treatment to proactive care. As Tullo et al. [120] note, such continuous monitoring systems allow for individualized comfort interventions, like cooling strategies during heat stress, which directly impact welfare outcomes. Furthermore, by reducing time to intervention, DTs decrease the duration and severity of animal discomfort, aligning closely with the Five Domains Model of Animal Welfare.

### 9.4. System-Wide Optimization

Beyond individual metrics, DTs contribute to systemic resilience by allowing adaptive decision-making under climate variability. Farms using such technologies can better navigate heatwaves, feed shortages, and disease outbreaks, ultimately leading to more stable production systems with lower environmental volatility. These systemic benefits are critical to the sustainability of livestock systems under increasing climate uncertainty [120].

## 10. Ethical Considerations

The implementation of DT systems in precision livestock nutrition raises complex ethical issues that require attention beyond their technological potential. A major concern involves data ownership and governance, especially as sensor-driven DTs collect continuous, high-resolution data streams from animals, equipment, and environmental systems. Without clear frameworks, data rights may default to technology providers rather than farmers, potentially undermining producer autonomy and creating dependencies [103]. In some real-world implementations, farm-generated data has been shared or sold to third parties, raising concerns over informed consent, value distribution, and long-term trust in digital agriculture platforms. Transparent contractual terms and farmer-inclusive data governance models are critical to prevent exploitation.

Additionally, the increased reliance on algorithmic decision-making in feeding systems can diminish the role of farmer intuition and experiential knowledge. This raises questions of agency and accountability, particularly when recommendations are not transparent and difficult to interpret due to black box AI models [122,123].The transparency of decision logic becomes even more critical in high-stakes scenarios, such as ration formulation, where nutritional errors can harm animal health. Ensuring that these systems are built on explainable AI (XAI) and include human-in-the-loop override capacity is essential to maintaining trust and responsible use [124].

Further, there are concerns of farms with limited access to connectivity, capital, or technical literacy, which may be excluded from DT-enabled benefits, exacerbating inequality across regions and operation scales [125]. Ethical deployment must therefore consider scalability, affordability, and inclusivity in DT design.

Finally, there are ecological and welfare trade-offs. DTs designed to optimize feed efficiency and productivity may unintentionally promote over-intensive systems, ignoring broader sustainability concerns such as methane emissions, nutrient runoff, or long-term soil degradation [126]. A responsible DT framework must integrate multi-objective goals that focus on economic optimization as well as environmental benefits and ethical treatment of animals. However, only a few studies have reported quantitative benefits, such as 70% energy saving through edge computation, and the representativeness remains unclear. Studies have not systematically assessed the energy footprint of DT deployments, making it difficult to benchmark their long-term sustainability. Future works should introduce energy auditing protocols and lifecycle analysis of digital infrastructure to ensure environmental accountability.

## 11. Data Governance and Energy Consumption in Digital Twin Implementation for Livestock Farming

Digital twin implementation in dairy nutrition faces critical data governance challenges that mirror broader controversies in agricultural technology, while energy consumption quantification remains severely underexplored. Real-world examples demonstrate alarming data governance failures, including John Deere’s monopolistic practices, where the Federal Trade Commission is investigating the company for using GPS data to track and discourage independent equipment repairs, creating what farmers describe as “monopolistic” activities that effectively tie all repairs to authorized dealers [127,128]. Similarly, the termination of The Climate Corporation’s partnership with Tillable highlights farmer concerns about data sharing, with CEO Corbett Kull emphasizing that “any data sharing through this integration was always going to be completely voluntary for farmers”; yet the partnership collapsed due to farmer backlash over data control issues [129]. These controversies exemplify broader patterns where agricultural technology providers accumulate massive amounts of farm data while farmers lose control over their most commercially sensitive information, with studies showing that 77.5% of farmers fear regulators might gain access to their private information without permission, and 82% have no idea what companies plan to do with their data [130,131].

Regarding energy consumption, the quantification gap is even more concerning—despite the transformative potential of digital twins, systematic energy auditing remains virtually absent from the livestock digital twin literature. While isolated studies like that of Menges et al. have reported 70% energy reductions at the edge [132], and building-based digital twins have demonstrated 23% energy savings [133], these figures are not representative due to the lack of standardized reporting protocols across agricultural digital twin deployments [134,135]. The few available studies focus primarily on edge computing benefits, showing that energy-aware nodes can dynamically adjust operating parameters and adopt power management techniques like sleep modes and voltage modification [136,137], yet comprehensive lifecycle assessments quantifying the total energy footprint of digital twin systems for dairy nutrition remain critically absent from current research, making sustainability claims impossible to verify systematically.

## 12. Current Trends and Future Directions

As digital twin technology evolves, emerging trends and innovations continue to expand its capabilities and applicability. They have focused on virtual monitoring and failure prediction and are now progressing toward self-learning, autonomous systems that evolve in real-time, adapt to contextual data, and enable closed-loop optimization across biological, mechanical, and social systems. This section explores how recent developments in artificial intelligence (AI), edge computing, simulation techniques, and integration with Industry 4.0 are shaping the future of DT systems. Additionally, it highlights open research gaps and proposes future research directions to address existing limitations and maximize the potential of DTs.

### 12.1. The Role of Artificial Intelligence and Machine Learning

One of the most significant shifts in DT systems is their growing reliance on AI/ML models for perception, prediction, and control. These are increasingly central to the development of intelligent, predictive digital twins.

DTs are evolving from deterministic rule-based systems to data-driven models that learn behaviors from experience. AI models such as LSTM, GANs, and transformers are being used to model complex time-series behaviors, forecast anomalies, and guide autonomous decision-making.

In livestock, AI-powered DTs for cattle caring and feeding behavior DTs [20] use CNNs to predict stress, optimize feed schedules, and detect anomalies. PsyDT [15] is a breakthrough in healthcare DTs, and Real2Sim2Real for autonomous vehicle simulation and deployment.

The recent literature [114] has emphasized the need for and importance of explainable and generalizable AI to improve trust, stakeholder-aligned model design, and usability across sectors.

This field is seeing the emergence of many novel AI architectures tailored for real-time, low-power DT applications. Neuromorphic computing [138] offers ultra-low energy spiking neural networks ideal for continuous behavioral inference on embedded devices. Similarly, TinyML [139] enables on-device learning and inference in resource-constrained environments, opening new possibilities for deploying AI models on low-cost sensors and microcontrollers. These innovations promise to make AI-powered DTs more accessible to small and mid-sized farms.

### 12.2. Edge Computing in Digital Twins

Edge computing is a major enabler for real-time DTs, reducing latency and bandwidth usage. Edge architectures are particularly effective in remote environments, such as dairy farms and smart greenhouses, where real-time decisions (e.g., feeding and irrigation) are critical.

In studies such as “AutoDRIVE” [25], real-time vehicular feedback loops are handled by onboard microcontrollers and fog gateways [140,141], pushing alerts upstream only when needed. “A Digital Twin for Smart Farming” [21] leveraged edge devices to support low-power, high-frequency data processing closer to the data source.

Challenges in deploying such architectures persist. Edge hardware is constrained by power, memory, and processing capabilities. Furthermore, managing distributed models across cloud and edge devices introduces complexity in terms of version control as well as security. However, the trade-off between responsiveness and centralization is increasingly favoring fog- and edge-based designs for real-world agricultural digital twins. These trade-offs emphasize the need for careful architectural design that balances latency, energy constraints, and deployment scalability, particularly in smaller dairy farm environments.

Several recent papers have emphasized the importance of hybrid deployment, where the edge node preprocesses data—noise filtering and anomaly detection—then forwards enriched, lower-volume payloads to cloud layers. This architecture not only reduces bandwidth consumption but also ensures more data privacy by minimizing the exposure of raw data. As 5G, LoRaWAN, and satellite internet become more accessible, the edge–cloud divide may become increasingly seamless, empowering more farms to deploy high-fidelity DTs without requiring industrial-grade infrastructure.

As edge computing matures, future paradigms such as quantum edge computing may offer breakthroughs in solving complex feed optimization and resource allocation tasks by leveraging quantum algorithms at the device level. While still largely experimental, such techniques could enable real-time decision-making over vast, non-linear search spaces. Additionally, the rollout of 6G networks is expected to bring ultra-low latency communication and high device density, improving the real-time performance and scalability of DT systems in remote agricultural environments.

### 12.3. Advanced Simulation Techniques

Simulation technologies are advancing toward greater realism and interactivity. Tools like Unity, Unreal Engine, and Simulink are being integrated with DT platforms for immersive training and high-fidelity testing environments. Neural implicit representations by Wang et al. [32] and procedural digital humans are being used in applications such as psychological counseling and rehabilitation modeling.

In disease and public health contexts, the use of GANs (Generative Adversarial Networks) and LSTM models to simulate alternate trajectories, as seen in COVID-19 twin studies [31], has implications for livestock health modeling. For example, stress-induced illness progression or nutritional deficiency onset could be pre-simulated in a virtual cow. This would allow farmers to test “what-if” scenarios before implementing costly or risky real-world interventions. These techniques allow more nuanced simulations of non-linear systems.

Despite their potential, simulation-based digital twins face considerable limitations. Generating realistic virtual environments require massive, labeled datasets that are usually unavailable or expensive to collect [142]. Synthetic simulation also raises concerns about model validity: if the virtual twin is trained on unrepresented data, its recommendations may be misleading. Moreover, integrating simulation engines with real-time operational data streams, especially in multi-modal input systems (vision, temperature, pH, and behavior) presents architectural challenges.

### 12.4. Industry 4.0 and the Future of Digital Twin Systems

The convergence of DTs with Industry 4.0 technologies such as IoT, cyber-physical systems, cloud platforms, and blockchain is driving a new era of intelligent infrastructure. Standardized DT toolchains and open-source frameworks (e.g., FIWARE [22] and IUMENTA [19]) are expected to reduce barriers to adoption. Research is also exploring integration with generative AI for autonomous system design and scenario exploration [143]. Key priorities moving forward include cross-domain interoperability, ethical governance, sustainability metrics, and democratized access for small and medium enterprises.

## 13. Conclusions

Digital twin (DT) technology represents a transformative innovation across diverse sectors, including manufacturing, healthcare, urban infrastructure, and, notably, agriculture and livestock management [144]. In particular, DTs have demonstrated substantial potential in precision dairy farming, significantly advancing the ability to monitor animal behavior, optimize individualized nutrition, and reduce environmental impacts such as greenhouse gas emissions. Central architectural advancements—comprising robust sensing capabilities, sophisticated data-driven modeling, and efficient connectivity through integrated edge–cloud frameworks—have positioned DTs as indispensable tools for real-time, predictive management of complex biological systems.

Despite these advances, several critical challenges remain unresolved. Interoperability across platforms continues to hinder the seamless integration of heterogeneous systems, particularly in rural settings where network infrastructure is limited. High implementation costs and the complexity of real-time data synchronization further complicate widespread adoption, especially among resource-constrained small and medium enterprises. Ethical concerns surrounding data privacy, governance, and transparent decision-making processes also persist, necessitating frameworks that balance technological advancement with stakeholder trust and animal welfare [145].

Looking toward 2030, the next frontier in digital twin development lies in the adoption of emerging computational paradigms that can enhance scalability, energy efficiency, and biological fidelity. Neuromorphic computing, which emulates biological neural networks to enable ultra-low-power inference, is particularly promising for continuous behavioral monitoring on edge devices. Quantum edge computing may one day offer the ability to solve complex multi-objective feed optimization and metabolic modeling problems in real time by exploring vast decision spaces beyond classical constraints. Additionally, advancements in 6G-enabled low-latency communication, bio-cyber interfaces for direct physiological sensing, and tinyML frameworks are expected to further enhance DT deployment in constrained farm environments. While many of these technologies remain nascent or experimental, their convergence presents a compelling roadmap for overcoming current trade-offs between performance, cost, and interpretability. Future research should explore how these paradigms can be adapted for rugged agricultural deployments and seamlessly integrated into real-time DT pipelines, setting a new trajectory for sustainable and intelligent livestock management.

Future research must prioritize the development of lightweight, interpretable AI models suitable for deployment in constrained farm environments. Progress toward standardized, cross-domain interoperability protocols and the establishment of open-source, modular DT frameworks is crucial to enable widespread, equitable adoption. Additionally, rigorous quantitative validation of DT technologies in active farming environments—with tangible metrics for performance, usability, and sustainability—is essential for transitioning from theoretical or experimental prototypes toward reliable, commercial-scale implementations.

Industry stands to benefit significantly from DT adoption through enhanced predictive maintenance, optimized operational processes, and the ability to deliver highly tailored products and services. In the agricultural sector, the integration of DTs promises substantial improvements in feed efficiency and significant reductions in methane emissions per animal [144], aligning closely with critical global sustainability objectives such as Zero Hunger, Responsible Consumption, and Climate Action outlined by the United Nations Sustainable Development Goals. Academia will play a pivotal role in this transformation, responsible for providing interdisciplinary education, developing open-access data repositories, and fostering collaborative platforms for robust validation and knowledge dissemination.

Ultimately, digital twins have transcended their conceptual origins, emerging as integral components of digital infrastructure essential for sustainable development and intelligent management of physical systems. To fully realize their promise, DT technologies must become universally accessible, overcoming barriers of complexity and cost, and thereby enabling sustainable, equitable, and intelligent solutions across sectors.

## Figures and Tables

**Table 1:** Summary of representative modeling techniques used in digital twin implementations across domains. Each entry specifies the model type, application domain, and its specific functional role within the digital twin pipeline.

Reference	Model Type	Application Domain	Purpose of DT
[31]	BiLSTM + GAN	Public Health	Time-series prediction of disease spread
[33]	CNN	Animal Husbandry	Feature extraction from images
[37]	XGBoost	Smart Farming	Feed conversion prediction
[34]	GAN	Behavioral Modeling	Generate training data for rare events
[21]	RL (Q-learning)	Crop and Livestock	Optimize irrigation and feeding
[4]	Physics + ML	Manufacturing	Fault detection and predictive control

**Table 2:** Computational characteristics of key functional modules in digital twin systems for precision livestock nutrition. The table compares algorithmic complexity, data throughput, processing environments, memory needs, and update frequency.

Component	Computational Complexity	Data Volume	Processing Location	Memory Requirements	Update Frequency	References
Feeding Behavior Analysis	O(n) for basic metrics O(n^2^) for pattern recognition	100 MB-1 GB/cow/day from accelerometers	Edge devices with ML capabilities	250 MB RAM for real-time processing	Every 5–15 min	[20,34]
Metabolic State Estimation	O(nlogn) for multi-parameter integration	10–50 MB/cow/day from ruminal sensors	Farm server with a dedicated GPU	2–4 GB RAM for model execution	Hourly updates	[41]
Feed Optimization Engine	O(n^3^) for multi-constraint optimization	5 MB/day for nutritional databases	Cloud service with distributed computing	8 GB RAM for population-level modeling	Daily or on-demand	[40]
Environmental Integration Module	O(n) for sensor fusion O(nlogn) for correlative analysis	1 GB/day for farm-level environmental data	Hybrid edge–loud architecture	1 GB RAM for contextual processing	Environmental triggers	[42]
Health Monitoring and Alerting	CNN and rule-based alerting	Biometric + behavior indicators (~500 MB/day)	Edge + central DB sync	1–2 GB RAM	Continuous/triggered	[39]

**Table 3:** Comparative analysis of deployment architectures in DTs for dairy farms. This table contrasts approximate characteristics of edge-only, cloud-only, and hybrid architectures. Values are based on representative implementations in the literature and are intended for qualitative comparison rather than precise benchmarking.

Architecture Type	Latency	Accuracy	Approx Cost	Scalability	References
Edge Only	~70% reduction compared to cloud	Not explicitly stated (edge-suitable CNN)	Low–moderate (Jetson-class devices)	Limited model complexity, high responsiveness	[39]
Cloud Only	High Latency	Handles complex optimization (e.g., genetic algorithms)	High (central compute + data transfer)	Centralized maintenance, bandwidth-dependent	[40]
Hybrid Models	Real-time inference at edge	>90% for behavior classification	Low: lightweight CNN + LSTM	Balanced with edge inference and point cloud integration	[20]

**Table 4:** Comparative assessment of modeling approaches used in digital twins for precision livestock nutrition. The table evaluates four major modeling paradigms across dimensions such as data requirements, computational efficiency, prediction accuracy, interpretability, and implementation complexity.

Modeling Approach	Mathematical Foundation	Data Requirements	Computational Efficiency	Prediction Accuracy	Interpretability	Implementation Complexity	References
Physics-Based Metabolic Models	Differential equations, compartmental models	Moderate: feed intake, pH, temperature, and milk yield	Moderate to high (depends on simulation resolution)	70–80% R2 for energy balance	High: clear causal relationships and biologically grounded	Medium: requires biological expertise and parameter calibration	[41]
Machine Learning (Neural Networks)	CNN for behavior, LSTM for temporal patterns, SVM	High: labeled accelerometer and intake data and image/video streams	Low for training, fast during inference	Up to 94% for intake prediction; 85–93% for behavior classification	Low: black box predictions	High: requires ML expertise for tuning and substantial labeled training data	[51,57]
Hybrid Models	Combined empirical and mechanistic	Moderate–high: sensor and historical data	Medium: modular components	85–90% under stable conditions (NIR + regression)	Moderate	High (multi-model integration)	[48,58]
Agent-Based Simulations	Individual cow agents with decision rules	Moderate: behavioral observations and historical patterns	Low for large herds, scales poorly	60–75% for individual behavior (better for aggregate patterns)	High: emergent behavior from clear rules	Medium: conceptually straightforward but difficult to parameterize accurately	[50,59]

**Table 5:** Overview of digital twin frameworks in agriculture and livestock. The table summarizes key research and implementation frameworks, highlighting their primary focus, applicable species or crops, and distinguishing technical features.

Framework	Focus Area	Species/Crop	Key Features
IUMENTA	Animal Behavior	Cows, Pigs	Modular, sensor-agnostic, real-time
SmartCow Data	Behavior Modeling	Dairy Cows	Annotated data for ML training
AgriLoRa	Feed and Integration	Dairy, Crops	RL + LoRa-based decision support
Digital Pig House	Housing Optimization	Swine	Simulated barn layout and climate
Horticulture DT	Root Zone Analysis	Greenhouse crops	Multi-sensor plant health DT
Bytes to Farm	Transferability	All	Industry-to-farm digital twin migration

**Table 6:** Comparative overview of digital twin applications across sectors. This table outlines the key functional roles, representative case studies, data sources, and domain-specific challenges in deploying digital twins in manufacturing, healthcare, smart cities, and agriculture.

Sector	Key Functions	Representative Works	Data Sources	Unique Challenges
Manufacturing	Predictive maintenance, virtual commissioning, optimization	Siemens DT [100], AutoDRIVE [25], Virtual Commissioning	Sensors (vibration, temp), PLCs, SCADA logs	Integration with legacy systems, cost of setup
Healthcare	Personalized medicine, mental health simulation, public health forecasting	PsyDT [15], COVID-19 DT [31], Precision Public Health	Wearables, EHRs, genomics, chat logs	Privacy, interpretability, regulation
Smart Cities	Traffic simulation, infrastructure monitoring, citizen feedback	CitySim [94], Herrenberg DT [92], Bogotá Smart City [93]	Drones, GISs, sensor grids, BIM	Data heterogeneity, real-time latency
Agriculture	Precision feeding, animal behavior modeling, crop forecasting	IUMENTA [19], AgriLoRa [21], SmartAgriFood [22]	RFID, GPS, bolus sensors, weathers, oil data	Rural connectivity, sensor failures, low-cost needs

**Table 7:** Estimated costs and infrastructure requirements for different digital twin deployment models in precision livestock farming. The table compares various deployment approaches based on initial investment, infrastructure needs, scalability, and potential barriers for small-scale farms.

Deployment Type	Initial Investment (USD)	Required Infrastructure	Scalability
Basic IoT Feed Sensors	USD 1000–USD 4000	Local data logger, wireless sensors	High
Cloud-Based DT System	USD 10,000–USD 18,000	Cloud API, stable internet, SaaS	Medium
Edge–AI Hybrid System	USD 15,000–USD 25,000	Edge device, local inference	High
Open-Source Modular DT	USD 2500–USD 6000	On-premises CPU, MQTT, OSS pipelines	Medium

**Table 8:** Technical barriers, current mitigation strategies, and research opportunities in digital twin deployment. This table outlines core technical limitations impacting system performance and summarizes both current solutions and emerging research directions as identified in the recent literature.

Technical Barrier	Impact on System Performance	Current Solutions	Technical Limitations	Research Opportunities	Reference
Rural Connectivity Limitations	Delayed model synchronization, loss of real-time actuation signals	Edge computing, LoRaWAN-based networks, asynchronous update scheduling	Limited ML computing capabilities at edge, fragmented data, unreliable sync	Federated edge inference, adaptive compression protocols, DT-aware synchronization	[115,116]
Sensor Data Quality Issues	False alarms, inaccurate behavior detection, temporal misalignment	Multi-sensor fusion, anomaly detection algorithms, sensor calibration pipeline	Sensor drift, energy limits, coverage variability	Transfer learning for sensor profiles, calibration-on-the-fly mechanisms	[20,37]
Computational Resource Constraints	Inability to run complex models locally, latency in cloud-only setups	Model compression, hardware-accelerated edge devices, scheduled analytics	Energy limits on devices, cloud cost, training offline	Lightweight CNN deployment, GPU virtualization for farms, Modular DL runtimes	[39,115,117]
Data Integration Heterogeneity	Errors in multi-modal fusion, inability to scale across farms	Semantic data layers (RFD, OWL), open APIs, ETL pipeline	Lack of standards, vendor-specific schemas, high maintenance	Auto-schema matching, distributed linked data infrastructure, blockchain-backed audit trails	[83,107]
Biological Variability Modeling	Poor generalizability of models across cows or herds	Baseline calibration per cow, hierarchical Bayesian models, dynamic parameter adjustment	Slow convergence, requires large initial dataset	Real-time Bayesian correction, embedded ensemble learning, biologically informed explainable AI	[118]
Legacy System Compatibility	DT cannot access historical or infrastructure-bound datasets	API wrappers, middleware bridges like MQTT, FIWARE adapters	Inconsistent metadata, slow update cycles, proprietary lock-in	Plug and play adapters, NLP-based data harmonization	[42]

## Data Availability

Not applicable.

## Abbreviations

The following abbreviations are used in this manuscript:

AI	Artificial Intelligence
CNN	Convolutional Neural Network
DDS	Data Distribution Service
DT	Digital Twin
DTaaS	Digital Twin as a Service
ETL	Extract Transform and Load
FMS	Farm Management Systems
GAN	Generative Adversarial Network
GECA	Global Edge Computing Architecture
GPU	Graphics Processing Unit
IoT	Internet of Things
KPI	Key Performance Indicators
LMIC	low- and middle-income countries
LP	Linear Programming
LSTM	Long Short-Term Memory
ML	Machine Learning
MQTT	Message Queuing Telemetry Transport
NLP	Natural Language Processing
NMB	normalized mean bias
OPC-UA	Open Platform Communications—Unified Architecture
RAM	Random Access Memory
RDF	Resource Description Framework
RL	Reinforcement Learning
RMSE	root mean square error
SVM	Support Vector Machine’
VFA	Volatile Fatty Acid
VIL	Vehicle-in-the-loop
WAN	Wide Area Network
WSN	Wireless Sensor Networks
XAI	Explainable Artificial Intelligence
XGBoost	Extreme Gradient Boost
